# Effects of sample handling and cultivation bias on the specificity of bacterial communities in keratose marine sponges

**DOI:** 10.3389/fmicb.2014.00611

**Published:** 2014-11-18

**Authors:** Cristiane C. P. Hardoim, Massimiliano Cardinale, Ana C. B. Cúcio, Ana I. S. Esteves, Gabriele Berg, Joana R. Xavier, Cymon J. Cox, Rodrigo Costa

**Affiliations:** ^1^Microbial Ecology and Evolution Research Group, Centre of Marine Sciences, University of AlgarveFaro, Portugal; ^2^Institute of Environmental Biotechnology, Graz University of TechnologyGraz, Austria; ^3^Department of Aquatic Microbiology, Institute for Biodiversity and Ecosystem Dynamics, University of AmsterdamAmsterdam, Netherlands; ^4^Department of Biology, Centre for Geobiology, University of BergenBergen, Norway; ^5^Plant Systematics and Bioinformatics, Centre of Marine Sciences, University of AlgarveFaro, Portugal

**Keywords:** holobiont, microbial cultivation, microbial diversity, pyrosequencing, symbiosis

## Abstract

Complex and distinct bacterial communities inhabit marine sponges and are believed to be essential to host survival, but our present-day inability to domesticate sponge symbionts in the laboratory hinders our access to the full metabolic breadth of these microbial consortia. We address bacterial cultivation bias in marine sponges using a procedure that enables direct comparison between cultivated and uncultivated symbiont community structures. Bacterial community profiling of the sympatric keratose species *Sarcotragus spinosulus* and *Ircinia variabilis* (Dictyoceratida, Irciniidae) was performed by polymerase chain reaction-denaturing gradient gel electrophoresis and 454-pyrosequecing of 16S rRNA gene fragments. Whereas cultivation-independent methods revealed species-specific bacterial community structures in these hosts, cultivation-dependent methods resulted in equivalent community assemblages from both species. Between 15 and 18 bacterial phyla were found in *S. spinosulus* and *I. variabilis* using cultivation-independent methods. However, *Alphaproteobacteria* and *Gammaproteobacteria* dominated the cultivation-dependent bacterial community. While cultivation-independent methods revealed about 200 and 220 operational taxonomic units (OTUs, 97% gene similarity) in *S. spinosulus* and *I. variabilis*, respectively, only 33 and 39 OTUs were found in these species via culturing. Nevertheless, around 50% of all cultured OTUs escaped detection by cultivation-independent methods, indicating that standard cultivation makes otherwise host-specific bacterial communities similar by selectively enriching for rarer and generalist symbionts. This study sheds new light on the diversity spectrum encompassed by cultivated and uncultivated sponge-associated bacteria. Moreover, it highlights the need to develop alternative culturing technologies to capture the dominant sponge symbiont fraction that currently remains recalcitrant to laboratory manipulation.

## INTRODUCTION

“The great plate count anomaly,” as introduced by Staley and Konopka (1985), describes the difference observed between the number of bacterial colony forming units (CFUs) grown on a culture medium and that of cells detected by microscopy for a given sample. This way, it was estimated that only 0.1–1.0% of the total bacterial cells in the environment could be accessed via standard cultivation approaches (Staley and Konopka, 1985). This result was supported by several studies of free-living (Kogure et al., 1979; Staley and Konopka, 1985; Amann et al., 1995) and host-associated bacterial communities (Friedrich et al., 2001; Webster and Hill, 2001). However, the plate count anomaly as originally described disregards the phylogenetic diversity of those CFUs grown on plates and uncultivated cells observed under the microscope (Donachie et al., 2007): the ratio CFU/microscopy cell counts, when directly used to describe “cultivation bias,” assumes even relative abundances between all microbial species or phylotypes that constitute the community. Despite this assumption, natural communities more often display uneven species abundances in which a few members dominate the assemblage followed by a majority of diverse, but rarer, species or phylotypes (Sogin et al., 2006; Gomes et al., 2010; Webster et al., 2010; Hardoim et al., 2013). Unfortunately, microbial ecologists have tended to overlook this pattern in the past when referring to our capacity to cultivate microbial species from the environment (Donachie et al., 2007). Several questions remain poorly understood, for instance, how does the diversity of readily cultivatable microorganisms compare with that resulting from cultivation-independent methods? Are we culturing the few dominant members of a community or preferentially the rare ones? Do communities resulting from cultivation-dependent and cultivation-independent methods share a majority of microbial phylotypes or are these communities divergent? Few studies have addressed these questions in detail (Donachie et al., 2007; Li et al., 2011; Sipkema et al., 2011; Montalvo et al., 2014), a situation which severely restricts our knowledge of the extent of the cultivatable microbiome diversity from natural habitats.

Marine sponges are reservoirs of microbial genetic and metabolic novelties. Up to 28 bacterial phyla, including candidate phyla, have been found in association with these animals (Simister et al., 2012). This conspicuous taxonomic diversification suggests functional variety, and several possible roles have been proposed for sponge symbionts upon interaction with their hosts (Taylor et al., 2007b; Webster and Taylor, 2012). Among them, the putative chemical defense enabled by biologically active compounds produced by sponge-associated bacteria is receiving considerable attention (Piel et al., 2004; Hochmuth and Piel, 2009; Siegl and Hentschel, 2010; Thomas et al., 2010b; Hentschel et al., 2012). Consequently, cultivating marine sponge bacteria is important from both a phylogenetic and biotechnological standpoint. Several attempts have been made to domesticate sponge-associated bacteria (Muscholl-Silberhorn et al., 2008; Esteves et al., 2013). Nevertheless, few studies assessed cultivation-dependent and -independent discrepancies in diversity surveys of the sponge microbiome (Sipkema et al., 2011; Montalvo et al., 2014).

The present study uses polymerase chain reaction-denaturing gradient gel electrophoresis (PCR-DGGE) and 454-pyrosequencing profiling to address the extent to which culturing and specific sample handling procedures affect the structure of bacterial communities in the keratose marine sponges (i.e., lacking mineral spicules and possessing an organic fibers skeleton instead) *Sarcotragus spinosulus* Schmidt 1862 and *Ircinia variabilis* Schmidt 1862. Previous studies suggest that these species harbor divergent bacterial communities and are the source of cultivatable bacteria with antimicrobial capacities (Hardoim et al., 2012; Esteves et al., 2013). However, the full diversity breadth of these microbial consortia and of their corresponding cultivatable fraction remains to be determined. We employed a strategy that circumvents the need to isolate single colonies during diversity surveys of cultivated bacteria and instead enables the direct comparison between bacterial community structures retrieved with cultivation-dependent and cultivation-independent methods. Although fewer bacterial species are usually recovered by cultivation than by cultivation-independent approaches, we hypothesized that cultivation would nevertheless represent the phylogenetic diversity of the communities assessed here to a higher extent than expected from CFU/microscopy count ratios. We also applied in-tube fluorescent *in situ* hybridization (FISH) of dominant sponge-associated bacteria to obtain the first insights into their localization and distribution in the two sponge species, and to compare the relative abundances of the bacterial symbionts estimated using sequencing and cell imaging technologies.

## MATERIALS AND METHODS

### SPONGE AND SEAWATER SAMPLING

Sampling took place at Galé Alta, Armação de Pêra (37° 04’ 09.6”N and 8° 19’ 52.1”W) off the coast of the Algarve, southern Portugal, in June 2010. Four specimens of *S. spinosulus* and *I. variabilis* (Demospongiae, Dictyoceratida, Irciniidae), and four samples of surrounding seawater (1 L each, about 1 m above sponge specimens), were collected in sterile Ziploc®; bags by scuba diving at about 15 m depth. Samples were placed in cooling boxes, transported to the laboratory (*c.* 2 h) and immediately processed as described below. Sponge species were identified based on macro- and microscopic morphological criteria coupled to molecular phylogenetic inference (Hardoim et al., 2012).

### CULTIVATION-INDEPENDENT AND -DEPENDENT TOTAL COMMUNITY DNA EXTRACTION

For each collected sponge specimen, three procedures of sample processing were undertaken prior to total community DNA (TC-DNA) extraction, hereafter called “direct,” “indirect,” and “plate washing” methods of sponge sample processing. The first two methods lead to cultivation-independent analysis of TC-DNA directly extracted from the sponge body (“direct” method) or from sponge-derived microbial cell pellets (“indirect” method). “Plate washing” involves extracting TC-DNA from washes of Marine Agar culture plates and is therefore a culture-dependent methodology for assessing the sponge-associated microbiome without having to purify and singularize colonies. Details of each procedure are given in Appendix S1 (Supplementary Material). TC-DNA extraction was performed with the UltraClean®; Soil DNA isolation kit (MO BIO, Carlsbad, CA, USA), according to the manufacturer’s protocol. The same kit was used for TC-DNA extraction from bulk seawater samples following Hardoim et al. (2012). Thus, 28 metagenome samples (four seawater replicates; four *I. variabilis* replicates handled with “direct,” “indirect,” and “plate washing” methods; and four *S. spinosulus* replicates handled with “direct,” “indirect,” and “plate washing” methods) were subjected to PCR-DGGE and 454-pyrosequencing bacterial community profiling as explained below.

### PCR-DGGE BACTERIAL COMMUNITY PROFILING

A nested PCR-DGGE approach targeting the V6 hypervariable region of the 16S rRNA gene was used to fingerprint the bacterial communities associated with both sponge species under the three methods of sample processing. The reaction mixture and thermal cycling for both reactions were as described by Hardoim et al. (2012), except for the concentration of primers (0.6 μM, **Table 1**). PCR-DGGE profiling was then performed using a PhorU-2 gradient system (Ingeny International, Goes, The Netherlands). Gel gradient, marker constituents, electrophoresis and staining procedures were described previously (Hardoim et al., 2012). Multivariate statistical analysis of PCR-DGGE fingerprints followed methods described elsewhere (Costa et al., 2006; Hardoim et al., 2009) and is detailed in Appendix S1.

**Table 1:** Polymerase chain reaction-denaturing gradient gel electrophoresis and 454-pyrosequencing primers used in this study.

Name	Sequence (5’–3’)	Usage	Reference
F27	AGAGTTTGATCMTGGCTCAG	First DGGE PCR	Weisburg et al. (1991)
R1492	TACGGYTACCTTGTTACGACTT	First DGGE PCR	Weisburg et al. (1991)
F984-GC	CGCCCGGGGCGCGCCCCGGGCGGGG CGGGGGCACGGGGGGAACGCGAAGAACCTTAC	Second DGGE PCR	Heuer et al. (1997)
R1378	CGGTGTGTACAAGGCCCGGGAACG	Second DGGE PCR	Heuer et al. (1997)
V4_titF	AYTGGGYDTAAAGNG	454-Pyrosequencing	http://pyro.cme.msu.edu/pyro/help.jsp#intro
V4_tit_R	TACNVRRGTHTCTAATYC	454-Pyrosequencing	http://pyro.cme.msu.edu/pyro/help.jsp#intro

### 454-PYROSEQUENCING BACTERIAL COMMUNITY PROFILING

A barcoded pyrosequencing approach was employed for in-depth analysis of bacterial community composition and diversity. A thorough description of (i) pyrosequencing sample preparation, (ii) data processing and (iii) analyses is provided in Appendix S1. Briefly, the V4 hypervariable region of the 16S rRNA gene was PCR-amplified using the Ribosomal Database Project primer set (**Table 1**), which generates amplicons of around 248 bp in length. Two PCR mixtures of 25 μL were prepared per sample, each containing ∼20 ng of template DNA. Each sample was tagged by unique 8-mer barcodes attached to the reverse primer. The amplicons were delivered for pyrosequencing on a 454 Genome Sequencer GS FLX Titanium platform (Roche Diagnostics Ltd, West Sussex, UK) at BIOCANT (Biotechnology Innovation Center, Cantanhede, Portugal). Raw data were processed using AmpliconNoise (Quince et al., 2011) and Galaxy (https://main.g2.bx.psu.edu/; Taylor et al., 2007a), enabling noise filtering (e.g., homopolymers), chimera removal, sequence sorting, and trimming. The Quantitative Insights Into Microbial Ecology (QIIME) software package (Caporaso et al., 2010) was then applied to the filtered data set for operational taxonomic units (OTUs) determination and taxonomic assignment, followed by the generation of a samples-OTUs table using customized scripts (Appendix S1). Data analyses encompassed (i) phylum- and class-level bacterial composition in individual and pooled samples, (ii) assessment of specific and shared bacterial symbionts across sample groups via OTU networks and Venn diagrams, (iii) estimates of symbiont richness (Chao1) and diversity (Shannon’s index) and (iv) multivariate analysis of OTU data. The latter was performed via (a) principal coordinate analysis (PCoA) of OTU profiles using the Unifrac metric within QIIME and (b) constrained ordination of OTU profiles and independent variables (i.e., seawater, sponge, sponge species, and sample processing methods) with the software package Canoco for Windows 4.5 using Hellinger-transformed OTU abundance data. Analyses (i) to (iv) were carried out using both full size (whole data set exploration) and size-normalized sample libraries. The analysis of full size libraries was used to determine the absolute number (and the identity) of all OTUs shared by and specific to each sample category (*n* = 7: seawater; *I. variabilis* under “direct,” “indirect,” and “plate washing” methods; and *S. spinosulus* under “direct,” “indirect,” and “plate washing” methods). The analysis of normalized libraries was applied in the quantitative comparison of bacterial richness, diversity and community structure between the sample categories. For size-normalized analyses, two depth thresholds were defined, 1236 and 3688 sequence reads per sample, which allowed the comparison of (i) all four replicate samples of both sponge species under the three methods of sample processing and triplicate seawater samples, and (ii) all sponge-derived libraries (seawater samples excluded), respectively. Similar analyses were performed for the unfiltered data set, disregarding chimera and noise removal procedures. Pyrosequencing data were deposited in the National Center for Biotechnology Information (NCBI) Sequence Read Archive (SRA) under the accession number SRP021445.

### IN-TUBE FLUORESCENT *IN SITU* HYBRIDIZATION AND CONFOCAL LASER SCANNING MICROSCOPY (FISH-CLSM)

To determine the spatial distribution and infer the abundance of sponge-associated bacteria at the micro-scale, in-tube FISH-CLSM was performed as described by Cardinale et al. (2008) with slight modifications (Appendix S1). For the detection of all bacteria, an equimolar mixture of Cy3-labeled EUB338, EUB338II, and EUB338III probes was used (Amann et al., 1990, **Table 2**). Samples were further hybridized with ALEXA488- or Cy5-labeled FISH probes specific for *Acidobacteria* (SS_HOl1400; Meisinger et al., 2007), *Alphaproteobacteria* (ALF968; Neef, 1997), and *Gammaproteobacteria* (Gam42a; Manz et al., 1992; **Table 2**). These taxa were selected based on their predominance revealed by 454-pyrosequencing. Taxon-specific abundances relative to total bacterial cell density were calculated by averaging the fraction of specifically stained cells from at least 15 randomly selected fields (confocal stacks) retrieved from three independent FISH experiments per specific probe.

**Table 2:** Fluorescent *in situ* hybridization probes used in this study.

Name	Sequence (5’–3’)	Target	Formamide (%)^1^	Reference
EUB338^2^	gctgcctcccgtaggagt	Most bacteria	20	Amann et al. (1990)
EUB338II^2^	gcagccacccgtaggtgt	*Planctomycetales*****	20	Daims et al. (1999)
EUB338III^2^	gctgccacccgtaggtgt	*Verrucomicrobiales*	20	Daims et al. (1999)
ALF968	ggtaaggttctgcgcgtt	*Alphaproteobacteria*	40	Neef (1997)
Gam42a	gccttcccacatcgttt	*Gammaproteobacteria*	40	Manz et al. (1992)
Gam42a competitor	gccttcccacttcgttt	*Betaproteobacteria*	40	Manz et al. (1992)
SS_HOL1400	ttcgtgatgtgacgggc	*Acidobacteria*	20	Meisinger et al. (2007)
NONEUB	actcctacgggaggcagc	–	^3^	Wallner et al. (1993)

*^1^Concentration of formamide for hybridizations at 43°C.*

*^2^Used in a mixture of equimolar concentrations.*

*^3^Used as negative control with the same formamide concentration as used for positive FISH.*

### TESTS OF SIGNIFICANCE

Homogeneity of variance tests were used to inspect the normal distribution of the richness and diversity measurements from PCR-DGGE fingerprints and 454-pyrosequencing. Analysis of variance (ANOVA) tested whether the mean values obtained for all sample groups (*n* = 7: seawater; *I. variabilis* under “direct,” “indirect,” and “plate washing” methods; and *S. spinosulus* under “direct,” “indirect,” and “plate washing” methods) were equal. A pairwise *t*-test which analyses the significance between groups was then carried out. Homogeneity of variance and ANOVA were also employed to compare 454-pyrosequencing relative abundances of the most dominant bacterial phyla and classes found across groups. The analyses were performed with the stat package in R programming (R Development Core Team, 2012). For both PCR-DGGE and 454-pyrosequencing data, Monte-Carlo permutations were performed to test whether the generated sponge symbiont profiles clustered according to the sample groups.

## RESULTS

### PCR-DGGE FINGERPRINTING OF BACTERIAL COMMUNITIES

**FIGURE 1:**
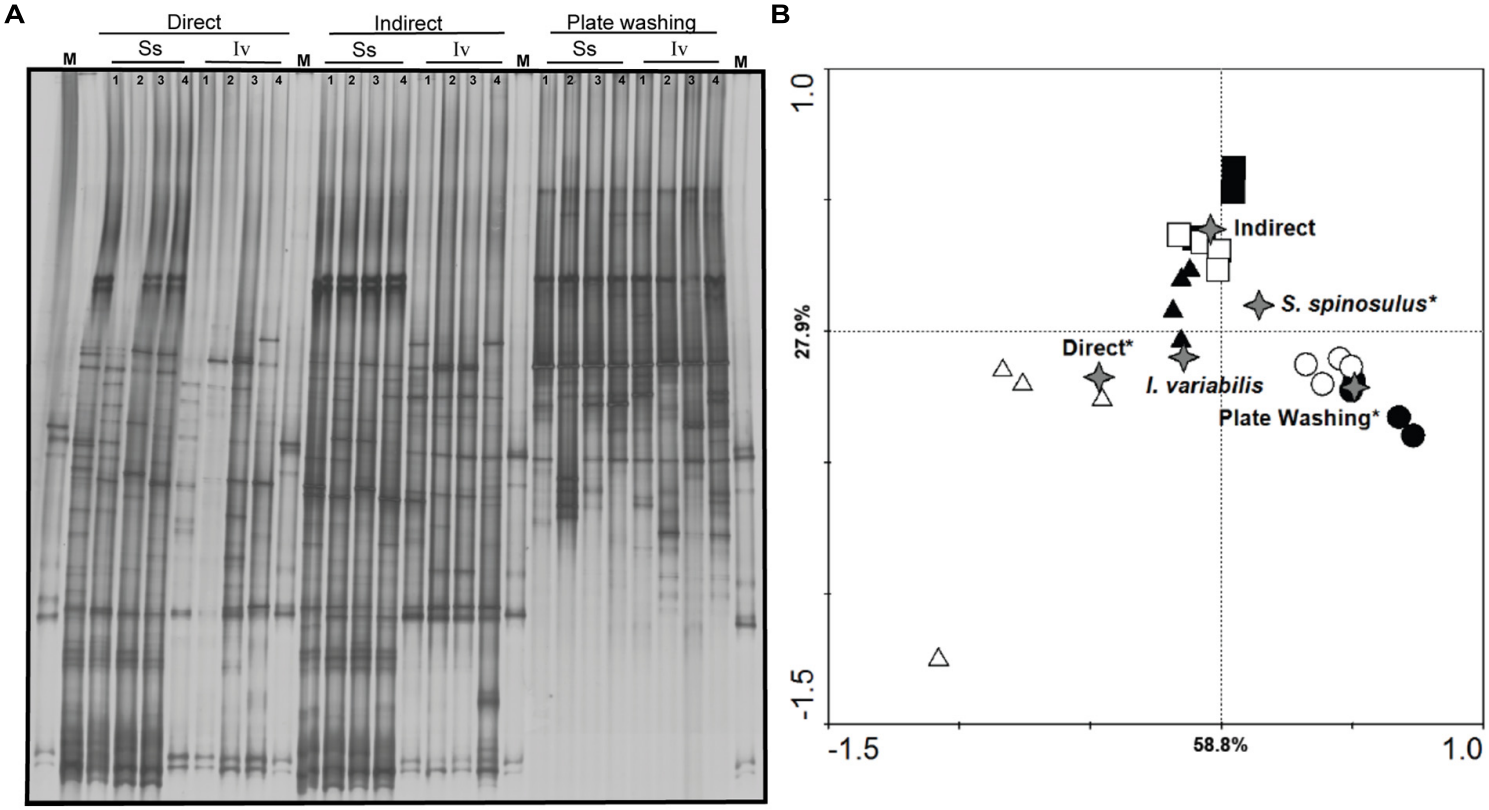
**Polymerase chain reaction-denaturing gradient gel electrophoresis 16S rRNA gene fingerprinting of bacterial communities associated with the marine sponges *Sarcotragus spinosulus (Ss)* and *Ircinia variabilis* (Iv).** Profiles obtained under the three methods of sample processing (“direct,” “indirect,” and “plate washing”) are shown **(A)** along with their corresponding ordination by redundancy analysis **(B)**. M: 16S rRNA gene marker used to control the DGGE run. Samples on the ordination diagram: ▲ – *S. spinosulus* “direct” processing, ■ – *S. spinosulus* “indirect” processing, ● – *S. spinosulus* “plate washing” processing, △ – *I. variabilis* “direct” processing, □ – *I. variabilis* “indirect” processing, ○ – *I. variabilis* “plate washing” processing. Labels displayed on the diagram axes refer to the percentage variations of PCR-DGGE ribotypes – environment correlation accounted for the respective axis. The “star” symbol represents the centroid positions of the canonical variables (i.e., sponge species and sample processing methods) in the diagram. Variables that significantly (*P* < 0.05) influenced bacterial community composition are highlighted with an asterisk.

The PCR-DGGE profiles of *S. spinosulus* obtained with cultivation-independent “direct” and “indirect” sample processing methods were visually very similar whereas much larger band variation was observed between *I. variabilis* fingerprints generated using the same methods (**Figure 1**). Profiles obtained for both sponge species via “plate washing” were different from those generated by cultivation-independent methods (**Figure 1A**). Ordination analysis of PCR-DGGE banding patterns suggested that both the host species and processing methods determined the structures of the surveyed symbiont communities (**Figure 1B**). A detailed description of PCR-DGGE results and a discussion on how they compare with 454-pyrosequencing data are given as Supplementary Material (Table S1, Appendix S2).

### 454-PYROSEQUENCING

#### Bacterial richness and diversity

**FIGURE 2:**
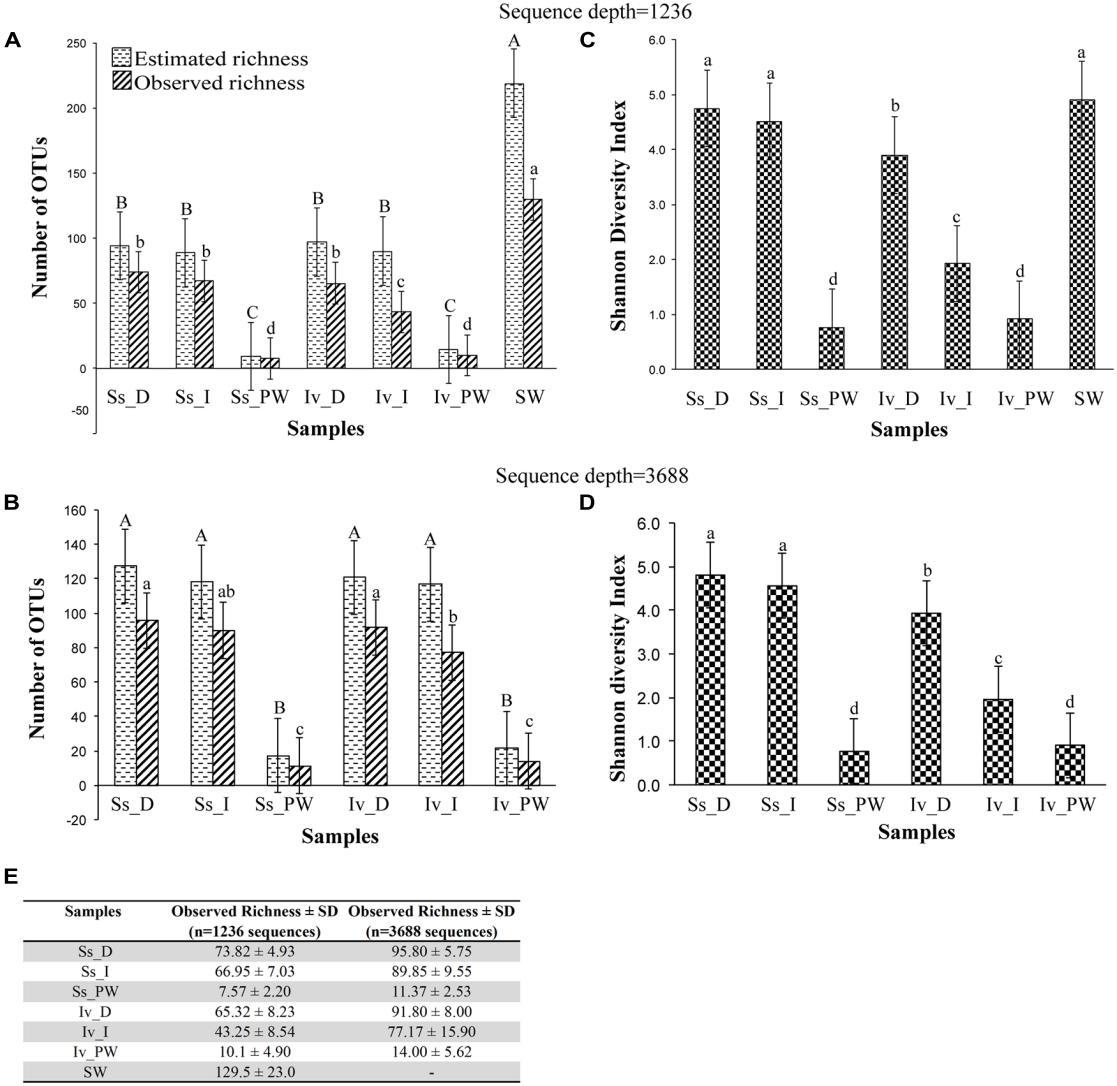
**Quantitative analysis of bacterial richness and diversity in marine sponges and seawater.** Observed and estimated (Chao1) richness measures **(A,B)**, Shannon diversity indices **(C,D)** and observed richness ± standard deviation **(E)** of bacterial OTUs are displayed using normalized depths of 1236 [includes seawater libraries, **(A,C,E)**] and 3866 [sponge libraries only, **(B,D,E)**] sequences per library. OTUs were determined at 97% 16S rRNA gene similarity. Values on bars are mean ± standard deviation of four replicates within each sample category, except for seawater where three replicates were used. Bars labeled with different letters represent statistically distinct sample categories in terms of richness and/or diversity values. In **(A,B)**, uppercase letters define differences in estimated richness across sample categories, whereas lowercase letters define differences in observed richness. Ss_D, *Sarcotragus spinosulus* under the “direct” processing method; Ss_I, *S. spinosulus* under the “indirect” processing method; Ss_PW, *S. spinosulus* under the “plate washing” processing method; Iv_D, *I. variabilis* under the “direct” processing method; Iv_I, *I. variabilis* under the “indirect” processing method; Iv_PW, *I. variabilis* under the “plate washing” processing method; SW, seawater.

A total of 237,773 16S rRNA V4-tag sequences passed preliminary filtering on the 454 apparatus. After sequence trimming and further quality filtering, 166,442 bacterial 16S rRNA gene V4-tag sequences were obtained and constituted the analytical data set. Sequences were assigned to 639 OTUs at a 97% similarity cut-off (**Table 3**). Considering all normalized sequence libraries (depth = 1236 sequences/sample), about twofold higher bacterial richness was observed in seawater than in sponge samples (**Figures 2A,E**). Bacterial richness increased significantly in both sponge species – and across all processing methods – when analyses were made with larger libraries (depth = 3668 sequences/sample). In this case, *S. spinosulus* and *I. variabilis* hosted from *c.* 80 to 95 bacterial OTUs/specimen under cultivation-independent methods (**Figures 2B,E**). Regardless of the sequence depth, two major trends were found across the data. First, a large reduction in bacterial symbiont richness and diversity was observed for both sponge species because of culturing (**Figures 2A–E**). Specifically, the number of bacterial OTUs detected using the “plate washing” method represented only 11.9 and 15.25% of the OTU richness recorded for *S. spinosulus* and *I. variabilis*, respectively, when using the “direct” method. Second, when cultivation-independent methods were compared, contrasting results were obtained for each sponge host. Whereas no difference in richness and diversity values was found for *S. spinosulus* treated with both the “direct” and “indirect” methods, handling of *I. variabilis* with the “indirect” method resulted in significant reduction of such estimates in this host (**Figures 2A–E**). Overall, Shannon diversity indices were not affected by the size of libraries used in the comparisons (**Figures 2C,D**), and seawater and *S. spinosulus* bacterial diversities obtained with culture-independent methods were of comparable magnitude (**Figure 2C**) in spite of the significantly higher bacterial richness detected in seawater (**Figure 2A**). Our sequencing effort (see **Table 3** for details) was found to encompass about 65, 81, and 84% of the estimated bacterial diversity in seawater, *S. spinosulus* and *I. variabilis* samples (“direct” method), respectively, while the diversity found in the cultivatable bacterial fraction was fully covered. Richness and diversity estimates for the unfiltered sequencing data are given in Figure S1 (Supplementary Material).

**Table 3:** Sequence data summary.

Sample type	Sample processing	N	454 filtering		454+AmpliconNoise filtering	
			Sequences	OTUs 97	Sequences	OTUs 97
*Sarcotragus spinosulus*	Direct	4	35,198	739	29,174	199
	Indirect	4	34,988	705	29,227	184
	Plate washing	4	27,497	247	24,547	33
*Ircinia variabilis*	Direct	4	29,503	671	24,222	215
	Indirect	4	25,899	541	22,686	225
	Plate washing	4	31,983	257	27,930	39
Seawater	n.a.	4	15,567	598	8656	329
Total	n.a.	28	200,635	1974	166,442	639

*N, number of replicate samples; n.a., not applicable.*

#### Community composition at the phylum level

**FIGURE 3:**
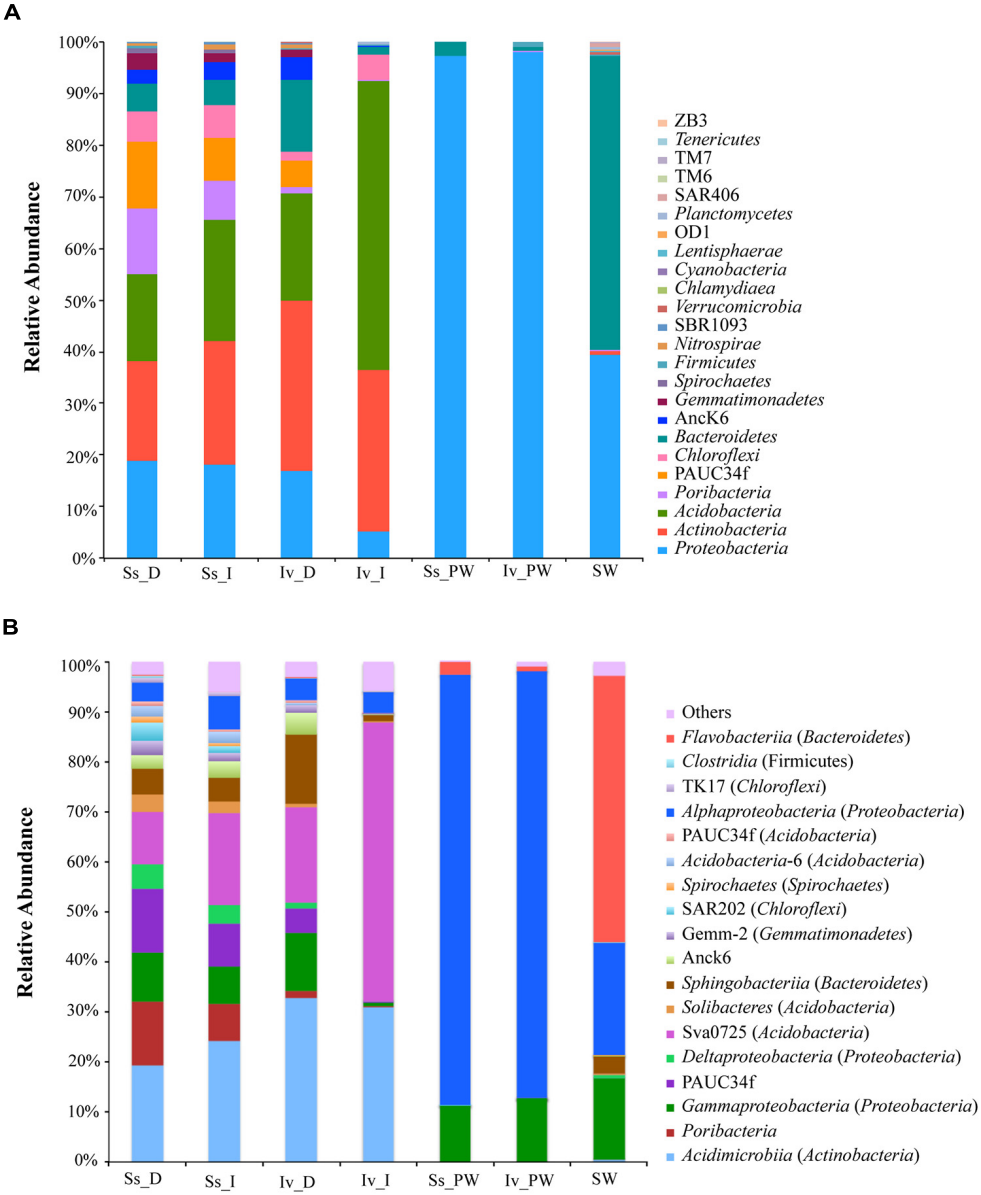
**Phylum- **(A)** and class-level **(B)** bacterial community composition in marine sponges and seawater.** Compositional data for *S. spinosulus* and *I. variabilis* handled with the “direct,” “indirect,” and “plate washing” processing methods are shown. Results obtained using pooled replicate samples (*n* = 4) within each sample category are displayed. Bacterial community composition in each replicate sample is shown as Supplementary Material (Figure S2). In **(B)** the top 18 bacterial classes are listed and all remaining taxa are labeled as “others.” Labeling of sample categories is as described in legend to **Figure 2**.

All major bacterial phyla detected in this study displayed significant shifts in relative abundance either as a function of the surveyed microenvironment (seawater *vs*. sponge), the host species (*S. spinosulus vs. I. variabilis*) or the sample processing method (“direct” *vs.* “indirect” *vs.* “plate washing”). Trends observed for the full quality-filtered data set (**Figure 3**), described below, were consistently reproduced when analyses made with size-normalized libraries were performed (Appendix S3, Supplementary Material). Seawater presented much lower bacterial richness at the phylum level than sponges (**Figure 3A**), it being largely dominated by *Bacteroidetes* (90 OTUs in 4918 sequences) and *Proteobacteria* (156 OTUs in 3401 sequences), which accounted for 57 and 40% of all sequences, respectively (**Figure 3A**, **Table 4**). By contrast, up to 21 bacterial phyla (438 OTUs in 157,786 sequences across all methods) could be detected in the sponge samples (**Figure 3A**), with each individual specimen usually hosting between 14 and 16 phyla (Figure S2, Supplementary Material). Cultivation-dependent bacterial communities from both sponges consisted mainly of *Proteobacteria* (>97% of all sequence reads) and were much reduced in phylum diversity when compared to cultivation-independent communities (**Figure 3A**). The most abundant sponge-associated phyla found using cultivation-independent methods were *Proteobacteria*, *Actinobacteria* and *Acidobacteria* (**Figure 3A**, Figure S2A). Considering only these methods, *Proteobacteria* was the most diverse phylum in sponges (188 OTUs in 16,127 sequences) followed by *Actinobacteria* (29 OTUs in 26,931 sequences) and *Acidobacteria* (27 OTUs in 30,346 sequences; **Table 4**). Differences in phylum relative abundances, without changes to within-phylum OTU richness, were observed among *I. variabilis* communities depending on which cultivation-independent method was used. For instance, higher abundance of *Acidobacteria* at the expense of much lower proportions of *Proteobacteria* and *Bacteroidetes* were retrieved with the “indirect” method when compared to the “direct” method (**Figure 3A**, Figure S2A, **Table 4**). In contrast, differences in relative abundances of phyla because of cultivation-independent, sample handling methods were negligible in *S. spinosulus* (**Table 4**).

**Table 4:** Number of OTUs and sequences per bacterial phylum across sample categories.

Phylum	Ss_D		Ss_I		Ss_PW		Iv_D		Iv_I		Iv_PW		Seawater	
	OTUs	seqs	OTUs	seqs	OTUs	seqs	OTUs	seqs	OTUs	seqs	OTUs	seqs	OTUs	seqs
*Acidobacteria*	15	4947	15	6806	0	0	15	5041	17	13,552	1	1	5	17
*Actinobacteria*	10	5674	10	7141	1	1	20	7976	21	6140	2	3	13	51
AncK6	1	802	1	896	0	0	2	1051	1	61	0	0	1	2
*Bacteroidetes*	16	1487	15	1305	3	678	21	3424	21	330	2	308	90	4918
*Chlamydiae*	0	0	0	0	0	0	0	0	3	8	0	0	0	0
*Chloroflexi*	35	1763	34	1902	1	1	18	411	19	1140	1	1	6	10
*Cyanobacteria*	0	0	0	0	0	0	3	6	3	8	0	0	1	1
*Firmicutes*	7	120	1	1	1	1	5	12	4	19	2	308	5	8
*Gemmatimonadetes*	8	879	7	503	0	0	7	364	5	17	0	0	1	7
*Lentisphaerae*	0	0	0	0	0	0	0	0	1	1	0	0	4	20
*Nitrospirae*	2	163	1	272	0	0	2	205	2	8	0	0	1	2
OD1	0	0	0	0	0	0	0	0	0	0	0	0	1	2
PAUC34f	4	3759	4	2634	0	0	4	1198	2	41	0	0	2	8
*Planctomycetes*	5	13	4	15	0	0	8	20	11	91	0	0	9	45
*Poribacteria*	6	3726	5	2137	0	0	5	318	4	14	1	1	2	11
*Proteobacteria*	80	5474	76	5312	27	23,866	101	4102	103	1239	30	27,308	156	3401
SAR406	0	0	0	0	0	0	0	0	0	0	0	0	14	82
SBR1093	1	32	1	120	0	0	1	84	1	2	0	0	0	0
*Spirochaetes*	2	319	3	162	0	0	1	2	1	2	0	0	1	1
*Tenericutes*	0	0	0	0	0	0	0	0	0	0	0	0	1	5
TM6	1	2	1	2	0	0	1	3	1	2	0	0	1	3
TM7	0	0	0	0	0	0	0	0	0	0	0	0	1	3
*Verrucomicrobia*	6	14	6	19	0	0	1	5	5	11	0	0	13	57
ZB3	0	0	0	0	0	0	0	0	0	0	0	0	1	2

Total	**199**	**29,174**	**184**	**29,227**	**33**	**24,547**	**215**	**24,222**	**225**	**22,686**	**39**	**27,930**	**329**	**8656**

*Values correspond to quality-filtered OTUs and sequences across the full data set. Ss, *Sarcotragus spinosulus*; Iv, *Ircinia variabilis*; D, “direct” method; I, “indirect” method; PW, “plate washing” method; seqs, sequence.*

Taxonomic classes within the most abundant phyla were assigned to OTUs when possible (**Figure 3B**). In seawater, *Flavobacteriia* (*Bacteroidetes*, 67 OTUs in 4576 sequences), *Alphaproteobacteria* (64 OTUs in 1803 sequences) and *Gammaproteobacteria* (62 OTUs in 1511 sequences) were the dominant classes (**Figure 3B**, Figure S2B). Comprising 113 OTUs in 14,270 sequences and 72 OTUs in 50,256 sequences across all processing methods, *Gammaproteobacteria* and *Alphaproteobacteria*, respectively, were the most abundant classes in the culture-dependent sponge bacterial communities. Using cultivation-independent procedures, however, their dominance was shared with several other classes, namely *Sphingobacteriia* (*Bacteroidetes*, 23 OTUs in 6478 sequences), *Acidimicrobiia* (*Actinobacteria*, 21 OTUs in 26,820 sequences), *Deltaproteobacteria* (20 OTUs in 2788 sequences), *Anaerolinea* (*Chloroflexi*, 14 OTUs in 2994 sequences) and Sva075 (*Acidobacteria*, 8 OTUs in 26,599 sequences; **Figure 3B**, Figure S2B).

#### Specificities and commonalities: shared and exclusive OTUs

**FIGURE 4:**
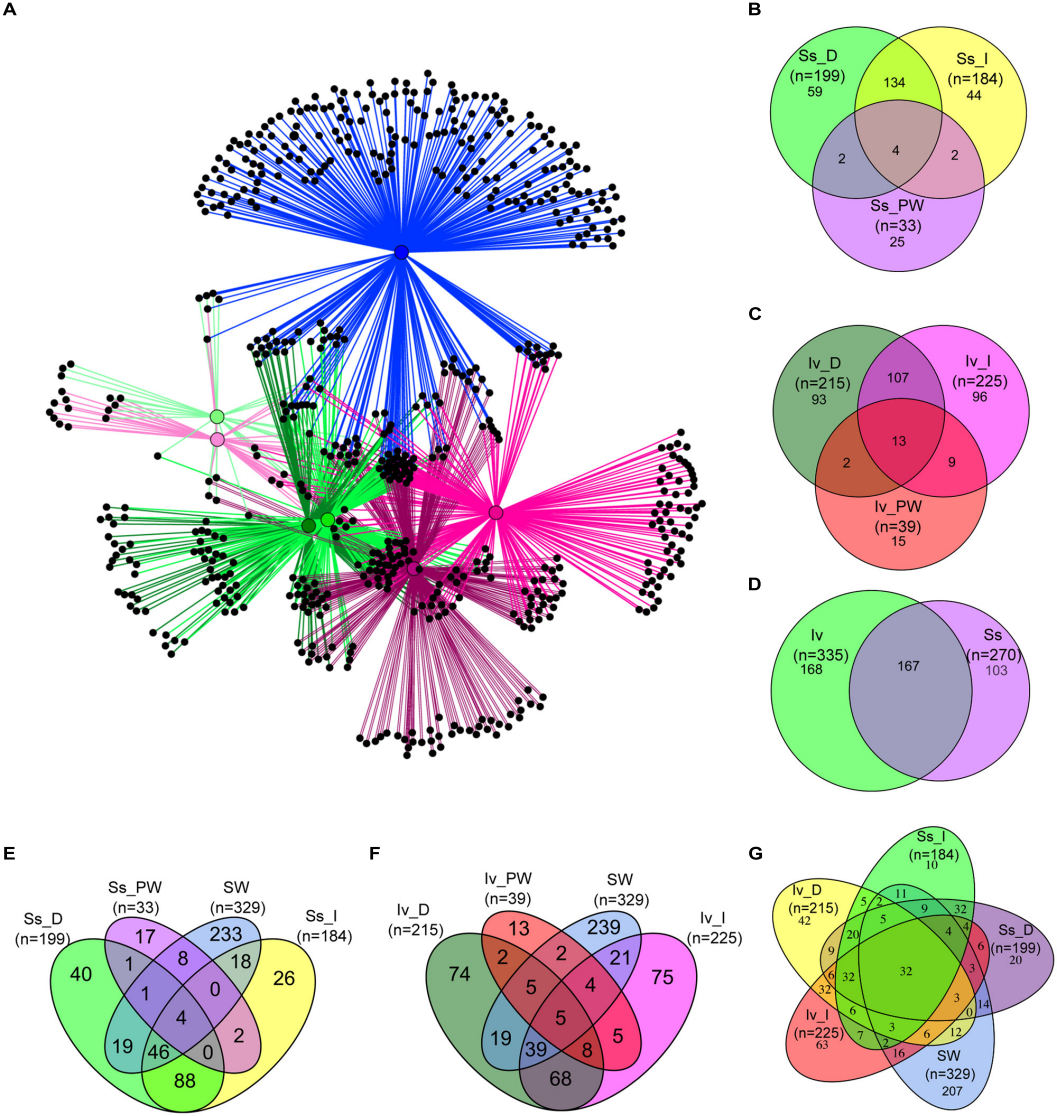
**Bacterial OTU networking and Venn diagrams.** All OTUs detected across all samples are included in the analysis. In the network diagram **(A)**, gradients of green correspond to *S. spinosulus* samples (light green: “plate washing” processing, green: “indirect” processing, dark green: “direct” processing), and gradients of pink to *I. variabilis* samples (light pink: “plate washing” processing, pink: “indirect” processing, dark pink: “direct” processing) along with their corresponding OTUs. Seawater samples and their corresponding OTUs are shown in blue. Venn diagrams **(B,C)** enumerate OTUs from *S. spinosulus* and *I. variabilis*, respectively, shared by and exclusive to the different methods of sample processing. Diagram **(D)** shows all OTUs detected in, shared by and exclusive to *S. spinosulus* and *I. variabilis* by merging the data obtained with the three processing methods. Diagrams **(E,F)** depict the degree of exclusiveness and sharedness between each species (under each of the three processing methods) and seawater, whereas diagram **(G)** compares marine sponge and seawater bacteriomes as determined by cultivation-independent methods only (i.e., “plate washing” sponge processing excluded). Labeling of sample categories is as described in legend to **Figure 2**. An equivalent analysis including only OTUs containing ≥50 sequences is shown as Supplementary Material (Figure S3).

Operational taxonomic units network analysis revealed that most bacterial OTUs found in seawater were specific to this environment, placing the bacterioplankton far apart from symbiotic communities (**Figure 4A**). Several bacterial OTUs exclusive to the “direct” and “indirect” methods were detected in *I. variabilis* community profiles, positioning *I. variabilis* samples processed with these methods farther apart from one another than the corresponding *S. spinosulus* samples in the network diagram (**Figure 4A**). Cultivation-dependent methods resulted in similar community compositions in both sponges, with only a few OTUs specific to each sponge species (**Figure 4A**). These trends were quantified using Venn diagrams (**Figures 4B–G**). Strikingly, only 4 and 13 bacterial OTUs were common to all three methods of sample processing in *S. spinosulus* and *I. variabilis*, respectively (**Figures 4B,C**). The proportion of OTUs specific to either of the cultivation-independent (“direct” and “indirect”) methods was indeed higher in *I. variabilis* than in *S. spinosulus* (**Figures 4B,C**). Despite the reduced bacterial diversity retrieved from the cultivation-dependent “plate washing” method, this approach led to the detection of several “cultivation-specific” OTUs not observed using cultivation-independent methods (**Figures 4B,C**). Dominant OTUs in the cultivatable community included the genera *Pseudovibrio*, *Vibrio*, *Shewanella*, *Aquimarina*, *Ruegeria,* and *Microbulbifer*. However, none of these taxa were ranked among the dominant OTUs captured by cultivation-independent methods. Unexpectedly, *Poribacteria*, *Chloroflexi* (SAR 202) and *Acidobacteria* (Sva0725) lineages were found in the cultivatable sponge community, however, each taxon was represented by one single OTU consisting of only one sequence read.

Irrespective of sample processing methods, 167 bacterial OTUs were found common to *S. spinosulus* and *I. variabilis*, while 103 and 168 bacterial OTUs were exclusively associated with each species, respectively (**Figure 4D**). For each sponge species, the most abundant “species-specific” OTU was affiliated with the *Bacteroidetes* phylum (class *Sphingobacteriia*). Bacterial OTUs found common to seawater and sponge communities from all processing methods numbered just 4 OTUs shared between *S. spinosulus* and seawater and five OTUs shared between *I. variabilis* and seawater (**Figures 4E,F**). These numbers rose to 32 bacterial OTUs shared by both sponge species with seawater when only cultivation-independent methods were considered (**Figure 4G**).

Analyses performed only with OTUs containing at least 50 sequences (i.e., “rare” symbionts discarded) showed that the number of otherwise considered “species-specific” or “method-specific” OTUs dramatically decreased (Figure S3, Supplementary Material). Notably, the community of “rare” OTUs was highly diverse in both sponge species, encompassing 163 and 236 OTUs found in *S. spinosulus* and *I. variabilis*, respectively, of which 86 and 160 OTUs were exclusive to each sponge species (Table S2, Supplementary Material). These OTUs comprised typical sponge-associated phyla encountered in the dominant symbiont pool such as *Acidobacteria*, *Actinobacteria*, *Chloroflexi*, *Proteobacteria* (*Alpha* and *Gamma* classes) and *Poribacteria* (Tables S2A,B). A thorough overview of the taxonomic affiliation of OTUs shared by or specific to the host species and processing methods surveyed in this study is provided in Appendix S4 (Supplementary Material). Highly congruent results with those reported above were obtained when network analysis was applied to size-normalized data sets (Appendix S5, Supplementary Material).

#### Ordination of bacterial OTUs

**FIGURE 5:**
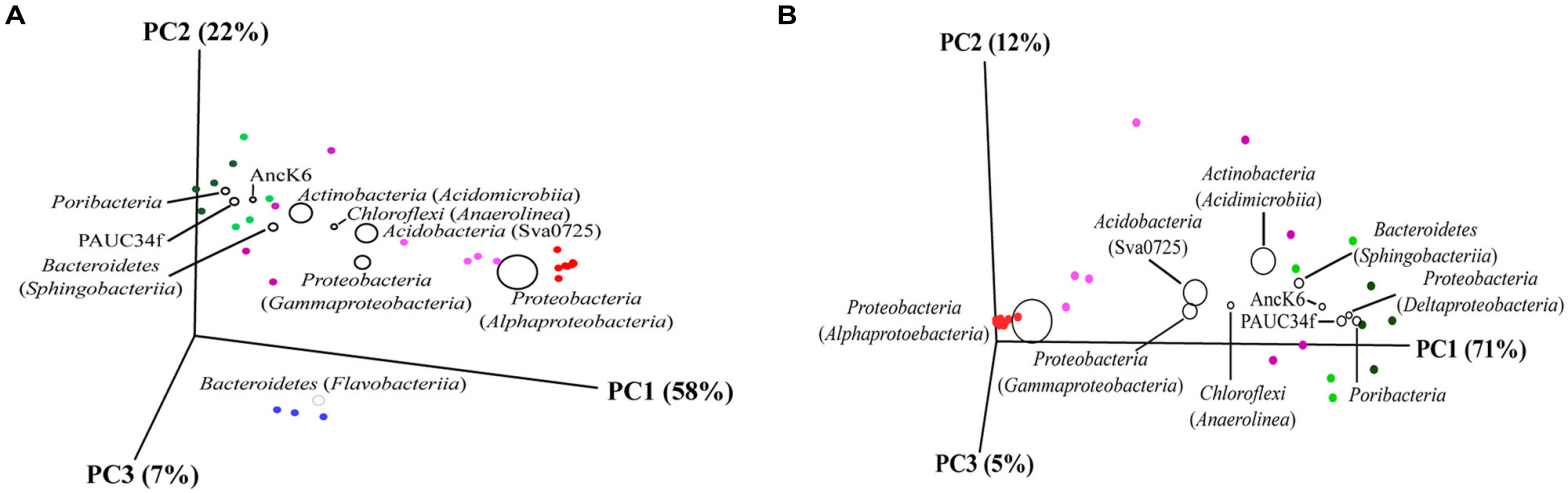
**Principal coordinates analysis of bacterial community profiles at the phylotype (OTUs) level.** Analyses including sponge and seawater samples **(A)** and sponge samples only **(B)** are shown, and were performed with normalized sequencing depths [1236 seqs/sample in **(A)** and 3668 seqs/sample in **(B)**] using the Unifrac metric. The ten most dominant bacterial taxa (at the phylum or class level) are plotted on both diagrams. Symbol sizes of bacterial taxa correspond to their respective, mean relative abundances across the whole data set. The position of bacterial taxa in the ordination space is determined by the correlation between their relative abundances and the sample categories defined in this study. Colored circles represent replicates within each sample category. Labeling of sample categories is as described in legend to **Figure 4**, except for both sponge species under the “plate washing” method, which are colored in red. Alternatively, constrained canonical ordination was performed using normalization of abundance data (and not of library size) to determine whether sample categories significantly influence variation in 16S rRNA gene profiling by 454-pyrosequencing. These analyses are shown as Supplementary Material (Figure S4).

For the first sequence threshold comparison (1236 sequences/ sample), two concise sample clusters could be visualized by PCoA: (i) all seawater replicates, and (ii) all sponge specimens processed with the “plate washing” method (**Figure 5A**). The remaining samples comprised all sponge replicates processed via cultivation-independent methods. The PCoA analyses showed a high similarity within *S. spinosulus* replicates treated with both cultivation-independent methods, while there was a lower correspondence between methods and higher individual-to-individual variability detected for *I. variabilis* samples (**Figure 5A**). After increasing the sequence depth by removing the seawater samples from the analysis (3688 sequences/sample, **Figure 5B**), the sharp dichotomy between sponge samples handled with cultivation-dependent and cultivation-independent methods persisted, whereas the divergence between *I. variabilis* specimens treated with cultivation-independent procedures became more apparent. For both sequence thresholds, the 3D plots were helpful in demonstrating correspondences between the samples and some of the most abundant bacterial phyla and/or classes, such as the prevalence of the *Alphaproteobacteria* in the culturable sponge fraction and the shift between *Flavobacteriia* and *Sphingobacteriia* as the prevalent *Bacteroidetes* class in seawater and sponge samples, respectively (**Figure 5**).

Canonical correspondence analysis (CCA) of the whole OTU data revealed that, collectively, independent variables (i.e., seawater, processing methods, and sponge species) could explain 46.8% of the total data set variation. The discrepancies between (i) cultivation-dependent and cultivation-independent methods, and (ii) sponge (all handling methods included) and seawater samples accounted for 41.8 and 41.4% of the explained variability, respectively (Figure S4, Supplementary Material). These values were considerably larger than the individual effects of the sponge species (*I. variabilis vs. S. spinosulus*, 12%) and cultivation-independent procedures (“direct” *vs.* “indirect,” 4.8%) on community data variation. The resulting CCA diagram distinguished seawater, sponges processed with cultivation-independent methods, and sponges processed with the cultivation-dependent method into three sample clusters (Figure S4A). Patterns of host species-specificity became evident when only *I. variabilis* and *S. spinosulus* specimens characterized by cultivation-independent methods were contrasted (Figure S4B), revealing that each sponge species held its own unique bacterial community. Finally, the shape of *I. variabilis* communities was significantly influenced by the cultivation-independent methods used (Figure S4C), however no such clustering was observed for *S. spinosulus* (Figure S4D).

### In tube FISH-CLSM

**FIGURE 6:**
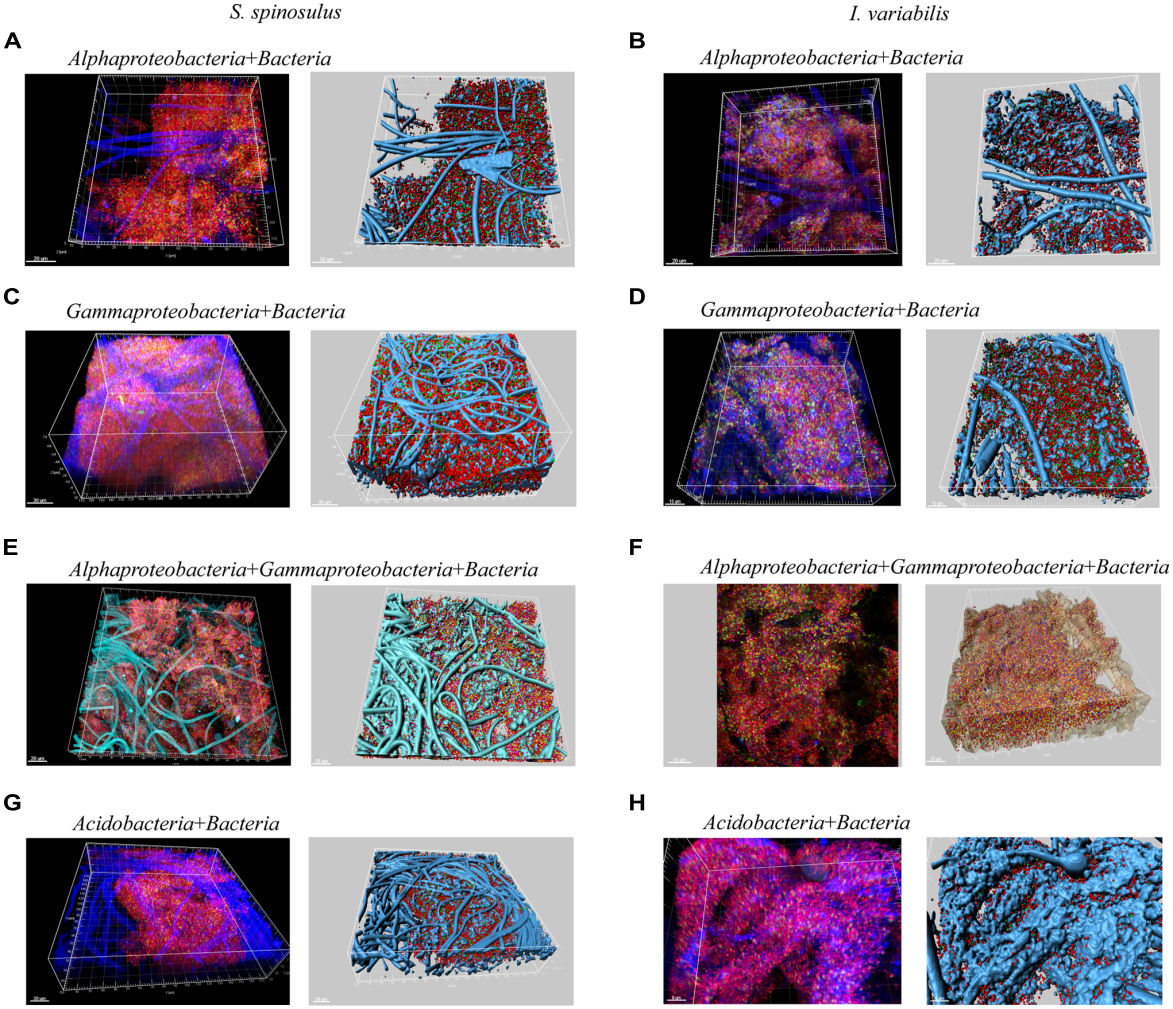
**Confocal laser scanning microscopy images of fluorescent *in situ* hybridization-stained bacteria in *S. spinosulus* and *I. variabilis*.** Volume rendering images (left in each panel) and their corresponding 3D reconstructions (right in each panel) are shown for hybridizations with the Cy3-labeled universal bacterial probe (red cells) coupled to ALEXA488- or Cy5-labeled group-specific probes targeting the *Alpha*- and *Gammaproteobacteria* classes and the phylum *Acidobacteria*. When solely used in combination with the universal probe **(A–D,G,H)**, cells of these taxonomic groups appear as yellowish cells in the volume rendering images and as green objects in the 3D reconstructions. For co-hybridizations including bacterial, alpha and gammaproteobacterial probes **(E,F)**, the latter two groups are represented by yellowish and pink cells, respectively, except in the 3D reconstruction of *I. variabilis* **(F)**, in which gammaproteobacterial cells appear in purple. Sponge background structure, vastly dominated by profuse spongin filaments, is shown overall in cyan or blue, except in **(F)** where it is displayed in semi-transparent brown.

The bacterial groups targeted by FISH were chosen to represent distinct patterns of abundance within the sequencing data set. Whereas *Proteobacteria* can be regarded as a “generalist” phylum dominant in seawater and sponge communities (both cultured and uncultured), *Acidobacteria* constitutes a “specialist” phylum with greater abundance within the unculturable sponge microbiome. The detected bacterial cells were mainly cocci found in between sponge cells, with no evidence for a taxon-dependent aggregation of bacteria within the sponge body. In all analyzed samples, bacterial cells were seldom found on spongin filaments. The high abundance of cells precluded discrete counting of cell numbers, and taxon abundance data relative to total bacterial coverage was retrieved instead. The relative abundance of *Alphaproteobacteria* in both sponge species was similar: 27.14% in *S. spinosulus* (**Figures 6A,E**) and 22.85% in *I. variabilis* (**Figures 6B,F**), whereas the *Gammaproteobacteria* were more abundant in *S. spinosulus* (21.41%, **Figures 6C,E**) than in *I. variabilis* (9.55%, **Figures 6D,F**). Both *Alphaproteobacteria* and *Gammaproteobacteria* were more abundant than *Acidobacteria* in *S. spinosulus* (9.09%, **Figure 6G**). In *I. variabilis*, the abundance of *Acidobacteria* (6.20%, **Figure 6H**) was similar to that of *Gammaproteobacteria* and lower than that of *Alphaproteobacteria*. Instead, *Acidobacteria* 16S rRNA gene tags accounted for 16.91 and 20.68% of the total communities in *S. spinosulus* and *I. variabilis*, respectively, surpassing numbers obtained for *Alpha*- (3.90% for *S. spinosulus* and 4.27% for *I. variabilis*) and *Gammaproteobacteria* (*S. spinosulus* 9.78% and *I. variabilis* 11.57%) in both sponge species according with 454-pyrosequencing.

## DISCUSSION

### SAMPLE HANDLING EFFECTS ON SPONGE BACTERIAL COMMUNITIES DEPEND ON THE HOST SPECIES

The extent to which existing methods of sample preservation, processing and DNA extraction may affect the structure of sponge symbiont communities is currently under-appreciated. Indeed, detailed analyses comparing sponge microbial community data retrieved with different methodologies are very scarce (see e.g., Hardoim et al., 2009; Simister et al., 2011). The “direct” processing method has been widely used to evaluate the diversity of microbial communities in marine sponges (Hentschel et al., 2002; Taylor et al., 2005; Lee et al., 2011; Hardoim et al., 2012) and is, moreover, the fundamental technique used in metatranscriptomic studies (Radax et al., 2012). By contrast, the “indirect” processing method, through which most of the sponge cells are eliminated, is used in metagenomic, metaproteomic, and single cell genomic surveys that target the sponge microbiota (Fieseler et al., 2006; Siegl and Hentschel, 2010; Thomas et al., 2010a; Siegl et al., 2011; Fan et al., 2012; Liu et al., 2012; Bayer et al., 2013). In the present study, both approaches led to similar bacterial diversity and composition results for *S. spinosulus*, but not for *I. variabilis*. In fact, we found that several OTUs consistently lost abundance while one OTU classified as *Acidobacteria* showed increased abundance in *I. variabilis* replicates processed with the “indirect” as compared to the “direct” method (see Appendix S6 for details), resulting in higher methodologically dependent symbiont community variability in this species than in *S. spinosulus*. This contrast may relate to the density of the collagenous filaments present in the mesohyl of these sponges, which is higher in *I. variabilis* making this species exceptionally tough and more difficult to tear or cut in comparison with other species in the Irciniidae family (Cook and Bergquist, 2002). Consequently, variability in the mechanical easy of bacterial cell detachment and disruption from the *I. variabilis* endosome matrix may have resulted in methodology-dependent, non-corresponding community structures retrieved from the same host. Rapid fingerprinting of sponge microbial communities by traditional methods such as PCR-DGGE or T-RFLP may still be an adequate means of assessing whether microbial cell enrichments usually prepared for metagenomics are representative of the community directly determined from the sponge body. Overall, the ability of each cultivation-independent processing method to accurately estimate the actual *in situ* sponge bacterial community may differ from species to species; therefore preliminary data acquisition to aid in the choice of methodology is advisable prior to in-depth analyses of sponge microbiome diversity and function.

### CONTRIBUTION OF ABUNDANT AND RARE OTUs TO HOST-SPECIFIC BACTERIAL COMMUNITY PROFILES

The degree of conservation of bacterial communities in marine sponges across host species, habitats and oceans has important implications for the management of marine genetic and metabolic resources given the status of these holobionts as the most prolific source of biologically active compounds in the oceans (Piel, 2004). Recent studies have suggested that these communities are host species-specific (Webster et al., 2010; Lee et al., 2011; Schmitt et al., 2012), contrary, in principle, to earlier studies which indicated microbiome conservation across sponge hosts and geographical locations (Hentschel et al., 2002). Through a detailed analysis of sympatric and co-familial sponges this study supports the increasing evidence for host-dependent symbiont community structures among phylogenetically related sponge species, acquired recently with the use of more traditional techniques (Erwin et al., 2012; Hardoim et al., 2012). Qualitatively, the pool of OTUs found exclusively in each species was largely circumscribed by “rare” symbionts: when OTUs deemed as less dominant were discarded from the analysis, the number of “host species-specific” bacterial phylotypes was drastically reduced whereas a strong signal for conservation of the more prevalent symbionts remained. We therefore propose that the views of host-conserved and host-specific bacterial communities in marine sponges are not mutually exclusive, but rather complementary facets of one single biological process. Indeed, the quantitative contribution of the so-called “rare” and often “species-specific” OTUs to the total bacteriome of the surveyed sponges was small (*c.* 1.9% of all analyzed sequence reads) despite their diversity. Host specificity was therefore found to be chiefly determined by full quantitative profiles including all community members, their corresponding abundance ranks and differences in abundance between host species. Interestingly, the phylum-level taxonomic composition of the uncultivated, “rare” sponge symbionts resembled that of the dominant symbionts. This could suggest maintenance of core, functional attributes across phylogenetically related microbes with varying abundances within the community, a mechanism that could confer functional stability to the marine sponge holobiont in face of changing conditions, be they host-induced or not. However, closely related bacterial species, and even strains within species, may also have widely differing functions that can result from the acquisition of traits via horizontal gene transfer, a well-documented phenomenon within the *Proteobacteria* (Costa et al., 2009; van Elsas et al., 2011). In light of the current evidence for abundant horizontal gene transfer potential (Thomas et al., 2010a) and whole genome differentiation among close bacterial relatives within the marine sponge microbiome (Esteves et al., 2013), the net contribution of intra- and inter-species bacterial diversity to the spectrum of functions in these communities remains to be understood.

### CULTURABLE SYMBIONTS ESCAPE THE MOLECULAR RADAR

It has been demonstrated that cultivation-independent methods often fail to detect bacteria isolated with culture-dependent procedures, raising concerns about the validity of cultivation-independent studies (Donachie et al., 2007). In our assay, cultivation of sponge-associated bacteria detected about 35–40 bacterial OTUs per species in comparison with 180–220 OTUs identified by cultivation-independent methods (see **Table 3** for details). These ratios surpass typical cultivability estimates based on CFU/microscopy cell count ratios, which in the case of our sponge specimens was in the range of 0.01–0.1% (Hardoim et al., 2012). However, about half of the OTUs recovered with the “plate washing” method escaped detection via cultivation-independent approaches, while the remainder was usually detected by the latter procedures in much lower numbers. These results indicate that marine agar culturing enriches for lower abundance symbionts that remain elusive to cultivation-independent methods. Because our methodology enabled direct comparisons between cultured and uncultured microbiomes through a standardized metagenomic DNA analysis pipeline, biases induced by DNA extraction, PCR amplification and colony picking-and-purification procedures are less likely to explain the abrupt differences observed between these communities in our survey. The hypothesis that the cultivatable sponge-associated bacteriome encompasses less abundant phylotypes that are considerably enriched during cultivation, by likely outcompeting more prevalent but less competent and/or slow-growing bacteria, must be assessed with protocols designed to detect rarer populations in the sponge community (Montalvo et al., 2014). In this regard, the in-tube FISH-CLSM approach used here to image highly ranked bacterial taxa may be a powerful tool to localize specific and less abundant symbionts in keratose sponges. In this study, FISH-CLSM and 454-pyrosequencing estimates of bacterial abundance were not always congruent. Variable target specificity and coverage between the techniques may have accounted for these differences, although the additional steps required for 454-pyrosequencing (cell detachment and lysis, DNA extraction and PCR) are expected to add more analytical biases compared to the direct FISH-CLSM method. Copy numbers are unlikely to explain the observed discrepancies. *Alphaproteobacteria* (more abundant in FISH-CLSM than in 454-pyrosequencing results) have an average of 2.4 copies of the ribosomal operon, whereas *Acidobacteria* (more abundant in 454-pyrosequencing) have an average of only 1.6 copies (Klappenbach et al., 2001). These results highlight the importance to use multiple methods for a more comprehensive analysis of symbiont abundance in marine sponges, as proposed elsewhere for other dense microbial settings such as soils (Ushio et al., 2014). Coupling nucleic acid sequencing (especially primer-less approaches, e.g., direct metagenome sequencing) and advanced imaging technologies seems an adequate strategy to be used in upcoming studies.

Although we did not anticipate the detection of *Acidobacteria*, *Chloroflexi,* and *Poribacteria* on culture plates, recent research supports their aerobic and heterotrophic growth capacities (Davis et al., 2011; Siegl et al., 2011; Kamke et al., 2013; see Appendix S7 – Supplementary Material – for a Discussion on *Poribacteria* results). Due to the very low number of sequences recovered, marine agar showed to be inappropriate for the future isolation of these organisms. Intriguingly, these and other lineages, such as *Kiloniella* sp. and *Ferrimonas* sp., could not be detected by a colony picking-and-isolation effort previously applied to our samples (Esteves et al., 2013). Beyond the comprehensiveness of the “plate washing-deep sequencing” procedure used here to characterize the cultivation-dependent communities, this outcome could be explained by the further possibility, enabled by this procedure, of uncovering strictly syntrophic microorganisms or micro-colonies that elude detection by the naked eye (Davis et al., 2011). Finally, the direct comparison between cultivation-dependent and -independent communities made in this study shows that traditional cultivation fails at capturing the prevalent bacterial associates of marine sponges. In this context, not only alternative medium recipes and incubation strategies are needed to enhance the cultivability of sponge symbionts in the laboratory, but improvements to our current methods to sample and analyze cultivated sponge microbiomes are desirable to extend our ability to assess the diversity and metabolic capacities of these and other symbiont cohorts in the future.

## AUTHOR CONTRIBUTIONS

Cristiane C. P. Hardoim, Ana I. S. Esteves, and Rodrigo Costa conceived and designed the study. Cristiane C. P. Hardoim, Ana I. S. Esteves, Massimiliano Cardinale, and Rodrigo Costa performed the laboratory experiments. Cristiane C. P. Hardoim, Ana C. B. Cúcio, Massimiliano Cardinale, Cymon J. Cox, and Rodrigo Costa analyzed the data. Rodrigo Costa, Gabriele Berg, Joana R. Xavier, and Cymon J. Cox contributed reagents, materials and analysis tools. Cristiane C. P. Hardoim, Massimiliano Cardinale, and Rodrigo Costa wrote the manuscript draft. All authors revised the draft, approved the final manuscript version and are accountable for all aspects of the work.

## Conflict of Interest Statement

The authors declare that the research was conducted in the absence of any commercial or financial relationships that could be construed as a potential conflict of interest.
