# Supplementary material for: Effects of sample handling and cultivation bias on the specificity of bacterial communities in keratose marine sponges
Source: Front Microbiol. 2014 Nov 18;5:611. doi: 10.3389/fmicb.2014.00611 (PMC4235377; doi:10.3389/fmicb.2014.00611)
Supplement: Supplementary file 1 [file Presentation_1.ZIP › Supplementary Material/Appendices_ File List and Legends.DOCX]

**Legends to Supplementary Material**

**Figure S1** Observed and estimated OTU richness and diversity for the unfiltered data set. Figure details are the same as provided in legend to **Figure 2**, except for the sequence-depth: 3640 [includes seawater libraries, panels (A) and (C)] and 4366 [sponge libraries only, panels (B) and (D)] sequences per library.

**Figure S2** Phylum- (A) and class-level (B) bacterial community composition in each replicate sample of *S. spinosulus*, *I. variabilis* and seawater. Figure details are the same as provided in legend to **Figure 3**.

**Figure S3** Network (A) and Venn diagrams (B-G) constructed for OTUs containing ≥ 50 sequences. Figure details are the same as described in legend to **Figure 4**.

**Figure S4** Constrained ordination analysis of bacterial communities profiled by 454-pyrosequencing. Ordination diagrams represent canonical correspondence analysis (CCA) embracing all sample categories (n = 7: seawater, *I. variabilis* with “direct”, “indirect” and “plate washing” methods, and *S. spinosulus* with “direct”, “indirect” and “plate washing” methods) (A); redundancy analysis (RDA) with both sponge species under cultivation-independent methods (B); RDA with *I. variabilis* under cultivation-independent methods (C), and RDA with *S. spinosulus* under cultivation-independent methods (D). Labelling of sample categories is as described in legend to **Figure 4**, except for both sponge species under the “plate washing” method, which are colored in red. Arrows indicate OTUs that were specific or enriched in *S. spinosulus* or *I. variabilis* under direct or indirect methods. Labels displayed on the diagram axes refer to the percentage variations of OTU abundance - environment correlation accounted for the respective axis. The ‘‘star’’ symbol represents the centroid positions of the canonical variables (i.e., sponge species and sample processing methods) in the diagram. Variables that significantly (*p* ***<*** 0.05) influenced bacterial community composition are highlighted with an asterisk.

**Table S1** Statistical analysis of PCR-DGGE band richness and diversity.

**Table S2** Taxonomic composition of rare OTUs found exclusively in *Ircinia variabilis* (A) and *Sarcotragus spinosulus* (B).

**Appendix S1** Detailed methodology.

**Appendix S2** PCR-DGGE fingerprinting results and discussion.

**Appendix S3** Phylum- (A) and class-level (B) bacterial community composition determined for normalized sample libraries (1236 sequences per sample). Details are as in legend to **Figure 3**.

**Appendix S4** Taxonomic affiliation of shared and specific OTUs across sponge species and methods.

**Appendix S5** OTU networks constructed for data sets normalized at 1236 (A) and 3688 (B) sequences per sample. Details are as in legend to **Figure 4**.

**Appendix S6** OTU vs. samples table generated from the full quality-filtered data set and OTU taxonomic assignments.

**Appendix S7** Distribution of *Poribacteria* OTUs across sample categories.
