# Supplementary material for: Effects of sample handling and cultivation bias on the specificity of bacterial communities in keratose marine sponges
Source: Front Microbiol. 2014 Nov 18;5:611. doi: 10.3389/fmicb.2014.00611 (PMC4235377; doi:10.3389/fmicb.2014.00611)
Supplement: Supplementary file 1 [file Presentation_1.ZIP › Supplementary Material/Appendix S1.DOCX]

**Appendix S1** Detailed methodology

**Sponge processing methods prior to total community DNA extraction**

In the “direct” method, 0.25 g (fresh weight) of internal sponge body was used for DNA extraction. For the “indirect” method, 2.5 g (fresh weight) of internal sponge body was ground with sterile mortar and pestle in 25 mL of Calcium/Magnesium Free Artificial Seawater (CMFASW) (Garson et al., 1998). The suspension was well mixed to produce a sponge-derived homogenate, which was further vortexed in a 50 mL sterile polypropylene tube and centrifuged at 500 *g* for 2 min to decant sponge cells and debris. The supernatant was transferred to a new 50 mL polypropylene tube and centrifuged at 9000 *g* for 30 min. The resulting microbial pellet was then used for DNA extraction. For the “plate washing” method, aliquots of homogenates prepared as above were serially diluted and plated onto Marine Agar (MA, Carl Roth GmbH**+**Co, Germany). After incubation for 5 days at 25^o^C, all colonies grown on a given plate were re-suspended in 3 mL of sterile artificial seawater (ASW; McLachlan, 1964) with the aid of a Drigalsky spatula and transferred into a 50 mL polypropylene tube. This procedure was performed for 6 MA plates per sample (triplicates of 10^-4^ and 10^-5^ dilutions), resulting in a 15 mL cultured cell suspension prepared for each sample. The suspension was thoroughly mixed and centrifuged at 9000 *g* for 30 min, after which the supernatant was discarded and DNA extraction was performed using the resulting cell pellet as starting material. As opposed to the “direct” and “indirect” methods, which enable cultivation-independent analyses of the sponge-associated microbiome, the “plate washing” method constituted a means of addressing, without purifying and singularizing colonies, the pool of sponge-associated bacteria culturable on MA.

**Analysis of PCR-DGGE fingerprints**

The software GelCompar II 5.1 (Applied Maths, Kortrijk, Belgium) was used to process the PCR-DGGE fingerprints as explained by Hardoim et al. (2009), delivering a contingency table species-samples containing the relative abundances of all bands in each profile. The table was used as input data for unconstrained (Principal Coordinate Analysis - PCA) and constrained (Redundancy Analysis - RDA) ordination analyses of PCR-DGGE fingerprints using Canoco for Windows 4.5 (Microcomputer Power, Ithaca, NY) as described in detail by Costa et al. (2006). PCR-DGGE band richness and diversity (Shannon-Wiener index) values were obtained for each sample using the Canoco software. These values were tested for significant differences between sample groups using the software package R (R Development Core Team 2012). Both datasets fitted the normal distribution and were subjected to factorial Analysis of Variance (ANOVA) coupled to Tukey´s Honestly Significant Differences (TukeyHSD) Test for pairwise comparisons.

**Preparation of samples for pyrosequencing**

Total community DNA samples were cleaned with Sephadex G50 columns (GE Healthcare Bio-Science AB, Uppsala, Sweden) and quantified with Nanodrop1000 (ThermoScientific, Delaware, USA). The V4 hypervariable region of the 16S rRNA gene was PCR-amplified using the primer set described in the pyrosequencing pipeline of the Ribosomal Database Project (**Table 1**), which generates amplicons of around 248bp in length. Two PCR mixtures of 25 µL were prepared per sample. Each mixture contained 1 µL of template DNA (~20 ng), 1X Bioline buffer (Bioline, London, UK), 0.2 mM deoxynucleoside triphosphates (dNTPs), 3.75 mM MgCl_2,_ 0.1 mg mL^-1^ of Bovine Serum Albumin (BSA), 5% (vol/vol) dimethyl sulfoxide (DMSO), 0.2 µM of forward and reverse primers, and 2.5U of *BioTaq*^TM^ DNA polymerase (Bioline, London, UK). Each sample was tagged by different 8-mer barcodes attached to the reverse primer. After initial denaturation at 94ºC for 4 min, 31 cycles of 30 sec at 94ºC, 45 sec at 44ºC, and 60 sec at 68ºC were performed, followed by a final extension for 10 min at 68ºC. The amplicons were delivered for pyrosequencing on a 454 Genome Sequencer GS FLX Titanium platform (Roche Diagnostics Ltd, West Sussex, UK) at BIOCANT (Biotechnology Innovation Center, Cantanhede, Portugal). Pyrosequencing data were deposited in the National Center for Biotechnology Information Sequence Read Archive (SRA) under the accession number SRP021445.

**Pyrosequencing data processing**

454-pyrosequencing raw data were processed using AmpliconNoise (Quince et al., 2011) for noise filtering (for instance, homopolymers), chimera removal, sequence sorting and trimming of maximum sequence length (≥ 260 bp). Trimming of minimum sequence length (≤ 150 bp, thus maintaining sequences between 150 and 260 bp) was achieved using the Galaxy software (https://main.g2.bx.psu.edu/; Taylor et al., 2007). The Quantitative Insights Into Microbial Ecology (QIIME) software package (Caporaso et al., 2010) was then applied to the filtered dataset for operational taxonomic units (OTUs) determination and taxonomic assignment, followed by the generation of a samples-OTUs table using customized scripts (see below). Briefly, OTUs were determined within QIIME at 97% sequence similarity using the UCLUST method (Edgar, 2010). Representative sequences of each OTU were picked using QIIME default parameters, and aligned employing Infernal (Nawrocki et al., 2009) against a STOCKHOLM file of pre-aligned sequences and secondary structures. Taxonomy assignment of representative sequences was performed using the BLAST taxonomy assigner method with the Greengenes 12_10 reference sequence database (http://greengenes.secondgenome.com/downloads/database/12_10). Based on the assigned taxonomy, a file was created with undesirable OTUs, that is, OTUs classified as chloroplasts, *Archaea* (1 OTU containing 2 sequences from seawater) and with no assigned taxonomy at the domain level (“no-blast hit”). These, along with OTUs represented by one single sequence (singletons), were removed from the samples-OTU table using a specific QIIME script (see below). A final OTU biom-format table was then created and used as input data for downstream analyses.

**Pyrosequencing data analysis**

Bar charts were created in QIIME describing OTU taxonomic assignments at the phylum and class levels for each sample as well as for sample categories in which replicates were pooled. For alpha- and beta-diversity metrics, libraries normalized for size were created by randomly picking sequences to avoid biases related with sequencing depth. Two sequence-depth thresholds were defined, 1236 and 3688 sequence reads, which allowed the comparison of (1) all four replicate samples of both sponge species under the three methods of sample processing plus triplicate seawater samples and (2) all the sponge-derived libraries, respectively. Shannon-Wiener diversity measures (Shannon, 1948b;a) and Chao1 richness estimates (Chao, 1984;Chao and Lee, 1992) were calculated for each sample in the QIIME environment (Caporaso et al., 2010). Multivariate analysis of community composition at the OTU level (97% sequence cut-off) was performed using the beta-diversity weighted Unique Fraction metric (UniFrac), which was applied to determine measures of (dis)similarity in bacterial community composition between samples (Lozupone and Knight, 2005). The distance matrix generated by weighted UniFrac was used for multivariate analysis by means of Unweighted Pair Group Method with Arithmetic means (UPGMA) clustering (Felsenstein, 2004) and Principal Coordinate Analysis (PCoA) (Krzanowski and Krzanowski, 2000). PCoA results were seen with the KiNG software package v. 2.21 (Chen et al., 2009). Jackknifed beta-diversity analysis was used to estimate the uncertainty in hierarchical clustering and PCoA plots of bacterial communities in all sample categories. 3D PCoA biplots were generated showing the 10 most abundant bacterial phyla for both sequence-depths and, when possible, abundant bacterial classes were also shown on ordination diagrams. Alternatively, to enable constrained multivariate analysis of the data with libraries not normalized for size, the OTU biom-format table was transformed into a tab-delimited table (see below), where OTU absolute abundances were converted into relative abundances. These were employed in constrained (RDA and Canonical Correspondence Analysis - CCA) ordination analyses with the software Canoco 4.5 (see Costa et al., 2006 for details) using Hellinger-transformed OTU abundance data. Similar statistical analyses were performed as well for the unfiltered dataset, disregarding chimera and noise removal procedures, following the tutorial available at the QIIME webpage with the minimum and maximum sequence length added to the “split_library.py” script. In this case, two normalized sequence-depths were also used, with 3540 and 4366 sequence reads for comparisons with and without seawater samples, respectively.

In order to determine the number of shared and specific OTUs across sample categories, an approach coupling OTU networks and Venn diagrams was employed. To this end, the four replicates within each sample category (n=7; seawater, *I. variabilis* with “direct”, “indirect” and “plate washing” methods, and *S. spinosulus* with “direct”, “indirect” and “plate washing” methods) were pooled. OTU networks and Venn diagrams were created (1) using all OTUs observed across the whole dataset and (2) taking only OTUs containing 50 or more sequences into account (i.e. “rare” phylotypes discarded). The inputs for OTU networks were generated using QIIME as described above (Caporaso et al., 2010) and analysed with Cytoscape version 2.8.3 (Smoot et al., 2011). The constructed networks approximate sample categories in the diagram space according to their OTU community composition, functioning thus as an exploratory proxy to cluster analysis. To determine the number of OTUs shared by and specific to each sample category in several combinations, diagrams were generated with the program “Venndiagram” as implemented in the software package R using default parameters (Chen and Boutros, 2011). To achieve this, the final OTU biom-format table obtained within QIIME was converted into a tab-delimited table (see below) where the list of OTU IDs found in each sample category was organized and used as input data.

**In-tube Fluorescent in situ Hybridization coupled with Confocal Laser Scanning Microscopy (FISH-CLSM)**

Small pieces of around 125 mm^3^ were cut from the inner part of the sponge specimens. Fixation of the sponge pieces was performed by incubation in 4% paraformaldehyde (PFA) at 4ºC for 6 h. Samples were then washed three times with ice-cold 1X phosphate buffered saline (PBS) to remove residual PFA, covered with an ice-cold mix (1:1) of 1X PBS and 96% ethanol, and stored at -20^o^C until further used. Prior to hybridization, 2-3 mm wide x 1 mm thick sections of the animals were prepared with forceps and scissors under magnifying glass, which showed to be an efficient method to maintain sponge internal structure. For the detection of all bacteria, an equimolar mixture of Cy3-labelled EUB338, EUB338II and EUB338III probes was used (Amann et al., 1990; **Table 2**). Samples were further hybridized with ALEXA488- or Cy5-labeled FISH probes specific for *Acidobacteria* (SS_HOl1400; Meisinger et al., 2007), *Alphaproteobacteria* (ALF968; Neef 1997), and *Gammaproteobacteria* (Gam42a; Manz et al., 1992) (**Table 2**). These taxa were selected based on their predominance revealed by 454-pyrosequencing. An unlabelled betaproteobacteria-specific probe (Gam42a-competitor; **Table 2**) was applied as a competitor together with probe Gam42a to avoid unspecific hybridization signals (Manz et al., 1992). A negative control was carried out in parallel with a non-sense FISH probe (NONEUB; Wallner et al., 1993; **Table 2**) labelled with the same fluorochromes used in the positive FISH. All FISH probes were purchased from Sigma-Aldrich, Vienna, Austria. In-tube FISH was performed as described by Cardinale et al. (2008), with some modifications as follows. The concentration of lysozyme was 0.5 mg mL^-1^, the ethanolic series was carried out with 50-80-96% ethanol solutions, hybridization was performed at 43ºC, washing buffer was pre-warmed at 44ºC, no incubation with the unspecific nucleic acid stain Sytox Blue took place and the sections were mounted with SlowFade^®^Gold antifade reagent (Invitrogen, Molecular Probes). CLSM was carried out with a Leica TCS SPE confocal microscope (Leica Microsystems, Mannheim, Germany). Fluorescent dyes Cy3, Cy5 and ALEXA488 were excited with 532, 635 and 488 nm laser beams, respectively; the emitted light was detected in the range of 545-634, 648-731 and 500-539 nm, respectively. To acquire the autofluorescence of the sponge internal structure (i.e. spongin fibres and filaments) an additional channel (excitation at 405 nm; emission range 425-462 nm) was applied. Photomultiplier gain and offset were optimised for every channel and field of view to improve the signal/noise ratio. Confocal stacks were acquired with a Leica ACS APO x 63 OIL CS objective (NA: 1.30) by applying a Z-step of around 0.6 µm. The software Imaris 7.0 (Bitplane, Zurich, Switzerland) was used to generate volume renderings and three-dimensional reconstructions.

**454-pyrosequencing analysis scripts**

1. **Pyrosequencing scripts used for the filtered dataset**

- Pyrosequencing analysis was carried out with the Quantitative Insights Into Microbial Ecology (QIIME) software package (Caporaso et al., 2010)
- To assign the pyrosequencing sequences to samples a mapping file was created with six columns: SampleID, BarcodeSequence, LinkerPrimerSequence, ReversePrimer, Treatment and Description. The reverse primer column was added because the barcode was attached to it. This file was saved as pyro.txt and checked for correct formatting with the script: *check_id_map.py -m pyro.txt -o mapping_output -v*
- The pyro.sff file was fragmented by running the script: *process_sff.py -i* pyro*.sff -f -o,* which generated the file needed for ampliconnoise (pyro.sff*.*txt)
- AmpliconNoise (Quince et al., 2011) was used to remove chimera and noise, to trim the sequences maximum length ≥ 260 bp and to assign sequences to sample IDs: *ampliconnoise.py -i pyro.sff.txt -m pyro.txt \ -o anoise_seqs --platform titanium -n 8 \--truncate_len 260*.
- Sequences ≤ 150 bp were removed from the fasta file created in the previous script. This was done with the software Galaxy (https://main.g2.bx.psu.edu/; Taylor et al., 2007). First the fasta file was uploaded (*Get data>Upload file from your computer>Browse>Execute*) and then sequences were trimmed (*FASTA manipulation*>*Filter sequences by length>minimum length>150*)
- The reverse primer was also removed from the fasta file produced in the previous step with *truncate_reverse_primer.py -f anoise_seqs_gal.fasta -m pyro.txt -o reverse_primer_removed/*; and the resulting file was renamed as *anoise_seqs_final.fna* and used for downstream analyses within QIIME pipeline
- Sequences sharing ≥ 97% similarity were grouped into operational taxonomic units (OTUs) using the default method (UCLUST; Edgar, 2010): *pick_otus.py -i reverse_primer_removed/anoise_seqs_final.fna -o picked_otus/*
- For each 97% OTU one representative sequence was selected with *pick_rep_set.py -i picked_otus/anoise_seqs_final_otus.txt -f reverse_primer_removed/anoise_seqs_*

*final.fna -o rep_set.fna*

- Representative OTU sequences were aligned with Infernal (Nawrocki et al., 2009) with *align_seqs.py -m infernal -i rep_set.fna -t seed.16s.reference_model.sto -o infernal_aligned/*
- Taxonomic assignment was carried out with the Greengenes database (http://greengenes.secondgenome.com/downloads/database/12_10) and the BLAST classifier: *assign_taxonomy.py -i rep_set.fna -r 97_otus.fasta -t 97_otu_taxonomy.txt -m blast*
- The infernal alignment was filtered with optimizations suggested by the QIIME team, for the definition of gap filter (-g) and base removal (entropy, -e) thresholds through the script *filter_alignment.py -i infernal_aligned/rep_set_aligned.fasta -s -g 0.80 -e 0.10 -o filtered_alignment/*
- Phylogenetic relationships between OTUs were then inferred with the alignment made previously: *make_phylogeny.py -i filtered_alignment/rep_set_aligned_pfiltered.fasta -o rep_phylo.tre*
- The OTU-samples biom-format table was created with: *make_otu_table.py -i picked_otus/anoise_seqs_final_otus.txt -t blast_assigned_taxonomy/rep_set_tax_*

*assignments.txt -o otu_table.biom*

- Based on the taxonomic assignment of the sequences, a file (remove.txt) was made with all undesirable OTUs, that is: those identified as chloroplasts and comprising sequences without assignment at domain level (“no blast-hit”). In addition, singletons (i.e. OTUs containing only one sequence) were also removed through the flag -n in *filter_otus_from_otu_table.py -i otu_table.biom -o otu_final.biom -n 2 -e remove.txt*
- The script *per_library_stats.py -i otu_final.biom* was used to check how many sequence reads were assigned to each library
- The OTU biom-format table was converted into a tab delimited table with *convert_biom.py -i otu_final.biom -o otu_final.from_biom.txt -b*, which was opened in an excel workbook and further used for the construction of Venn diagrams (Chen and Boutros, 2011) and constrained ordination analyses with Canoco for Windows 4.5
- An OTU network was created with *make_otu_network.py -m pyro.txt -i otu_final.biom -o otu_network* and analyzed with Cytoscape (Smoot et al., 2011)
- Taxonomy summary files at several levels were generated with *summarize_taxa_through_plots.py -i otu_final.biom -o taxa_summary -m pyro.txt*
- Before running the alpha diversity metric, the Shannon index of diversity was included with *echo “alpha_diversity:metrics shannon,chao1,observed_species, PD_whole_tree” > alpha_params.txt* and then the alpha diversity script *alpha_rarefaction.py -i otu_final.biom -m pyro.txt -o wf_arare/-p alpha_params.txt -t rep_phylo.tre* was applied
- For beta diversity metrics, two sequence thresholds were selected allowing the comparison of (1) all libraries (n=1236) and (2) only sponge-derived libraries (n=3688) with the following script: *beta_diversity_through_plots.py -i otu_final.biom -o bdiv_even1236/ -t rep_phylo.tre -m pyro.txt -e 1236 --color_by_all_fields* and *beta_diversity_through_plots.py -i otu_final.biom -o bdiv_even3688/ -t rep_phylo.tre -m pyro.txt -e 3688 --color_by_all_fields*
- Jackknifed beta diversity was used to estimate the uncertainty in Principal Coordinate Analysis (PCoA) and hierarchical clustering for both sequence thresholds with the script: *jackknifed_beta_diversity.py -i otu_final.biom -t rep_phylo.tre -m pyro.txt -o wf_jack_blast -e 1236* or *-e 3688*
- In addition a bootstrapped tree was created for both sequence thresholds (n=1236 and n=3688) with: *make_bootstrapped_tree.py -m wf_jack /weighted_unifrac/upgma_cmp/master_tree.tre -s wf_jack /weighted_unifrac/upgma_cmp/jackknife_support.txt -o wf_jack /weighted_unifrac/upgma_cmp /jackknife_named_nodes.pdf*
- Finally, a 3D PCoA plot was generated for both sequence thresholds with the centroid positions of the 10 most abundant phyla with: *make_3d_plots.py -i bdiv_even1236/weighted_unifrac_pc.txt -m pyro.txt -t taxa_summary/otu_final_L3.txt --n_taxa_keep 10 -o 3d_biplot10* and *make_3d_plots.py -i bdiv_even3688/weighted_unifrac_pc.txt -m pyro.txt -t taxa_summary/otu_final_L3.txt --n_taxa_keep 10 -o 3d_biplot10*

1. **Pyrosequencing scripts used for the unfiltered dataset**

- Pyrosequencing analysis was carried out with QIIME (Caporaso et al., 2010)
- A mapping file was generated with six columns: SampleID, BarcodeSequence, LinkerPrimerSequence, ReversePrimer, Treatment and Description, and checked with *check_id_map.py -m pyro.txt -o mapping_output -v*
- *process_sff.py -i* pyro*.sff -f -o,* which generated the files needed for the next script
- *split_libraries.py -m pyro.txt -f pyro.fna -q pyro.qual -o split_library_output/ -b hamming_8 -l 150 -L 260 -z ‘truncate_only’*, where -l and-L are the minimum and maximum sequence length, respectively
- *pick_otus.py -i split_library_output/seqs.fna -o picked_otus/*
- *pick_rep_set.py -i picked_otus/seqs_otus.txt -f split_library_output/seqs.fna -o rep_set.fna*
- *align_seqs.py -m infernal -i rep_set.fna -t seed.16s.reference_model.sto -o infernal_aligned/*
- *assign_taxonomy.py -i rep_set.fna -r 97_otus.fasta -t 97_otu_taxonomy.txt -m blast*
- *filter_alignment.py -i infernal_aligned/rep_set_aligned.fasta -s -g 0.80 -e 0.10 -o filtered_alignment*
- *make_phylogeny.py -i filtered_alignment/rep_set_aligned_pfiltered.fasta -o rep_phylo.tre*
- *make_otu_table.py -i picked_otus/seqs_otus.txt -t blast_assigned_taxonomy/rep_set_tax_assignments.txt -o otu_table.biom*
- *filter_otus_from_otu_table.py -i otu_table_blast.biom -o otu_final.biom -n 2 -e remove.txt*
- *per_library_stats.py -i otu_final.biom*
- *convert_biom.py -i otu_final.biom -o otu_final.from_biom.txt -b*
- *make_otu_network.py -m pyro.txt -i otu_final.biom -o otu_network*
- *summarize_taxa_through_plots.py -i otu_final.biom -o taxa_summary -m pyro.txt*
- *echo “alpha_diversity:metrics shannon,chao1,observed_species, PD_whole_tree” > alpha_params.txt* and then the alpha diversity script *alpha_rarefaction.py -i otu_final.biom -m pyro.txt -o wf_arare/-p alpha_params.txt -t rep_phylo.tre*
- *beta_diversity_through_plots.py -i otu_final.biom -o bdiv_even3640/* or *-o bdiv_even4366/ -t rep_phylo.tre -m pyro.txt -e 3640 or -e 4366 --color_by_all_fields*
- *jackknifed_beta_diversity.py -i otu_final.biom -t rep_phylo.tre -m pyro.txt -o wf_jack_blast -e 3640* or *-e 4366*
- *make_bootstrapped_tree.py -m wf_jack/weighted_unifrac/upgma_cmp/master_tree.tre -s* *wf_jack/weighted_unifrac/upgma_cmp/jackknife_support.txt -o wf_jack/weighted_unifrac/upgma_cmp/jackknife_named_nodes.pdf*
- *make_3d_plots.py -i bdiv_even3640/weighted_unifrac_pc.txt* or *-i bdiv_even4366/weighted_unifrac_pc.txt -m pyro.txt -t taxa_summary/otu_final_L3.txt --n_taxa_keep 10 -o 3d_biplot10*

**References**

Amann, R.I., Binder, B.J., Olson, R.J., Chisholm, S.W., Devereux, R., and Stahl, D.A. (1990). Combination of 16S rRNA-targeted oligonucleotide probes with flow cytometry for analyzing mixed microbial populations. *Appl. Environ. Microbiol.* 56, 1919-1925.

Caporaso, J.G., Kuczynski, J., Stombaugh, J., Bittinger, K., Bushman, F.D., Costello, E.K., et al. (2010). QIIME allows analysis of high-throughput community sequencing data. *Nat. Methods* 7, 335-336. doi:10.1038/nmeth.f.303

Cardinale, M., Vieira De Castro Jr, J., Müller, H., Berg, G., and Grube, M. (2008). *In situ* analysis of the bacterial community associated with the reindeer lichen *Cladonia* *arbuscula* reveals predominance of *Alphaproteobacteria*. *FEMS Microbiol. Ecol.* 66, 63-71. doi:10.1111/j.1574-6941.2008.00546.x

Chao, A. (1984). Nonparametric-estimation of the number of classes in a population. *Scand. J. Statist.* 11, 265-270.

Chao, A., and Lee, S.M. (1992). Estimating the number of classes via sample coverage. *J. Am. Stat. Assoc.* 87, 210-217.

Chen, H., and Boutros, P.C. (2011). VennDiagram: a package for the generation of highly-customizable Venn and Euler diagrams in R. *BMC Bioinformatics* 12. doi:10.1186/1471-2105-12-35

Chen, V.B., Davis, I.W., and Richardson, D.C. (2009). KiNG (Kinemage, Next Generation): A versatile interactive molecular and scientific visualization program. *Prot. Sci.* 18, 2403-2409. doi: 10.1002/pro.250

Costa, R., Salles, J.F., Berg, G., and Smalla, K. (2006). Cultivation-independent analysis of *Pseudomonas* species in soil and in the rhizosphere of field-grown *Verticillium dahliae* host plants. *Environ. Microbiol.* 8, 2136-2149. doi:10.1111/j.1462-2920.2006.01096.x

Edgar, R.C. (2010). Search and clustering orders of magnitude faster than BLAST. *Bioinformatics* 26, 2460-2461. doi:10.1093/bioinformatics/btq461

Felsenstein, J. (2004). *Inferring phylogenies*. (Sinauer Associates/Sunderland, Massachusetts).

Garson, M.J., Flowers, A.E., Webb, R.I., Charan, R.D., and Mccaffrey, E.J. (1998). A sponge/dinoflagellate association in the haplosclerid sponge *Haliclona* sp.: cellular origin of cytotoxic alkaloids by Percoll density gradient fractionation. *Cell Tissue Res.* 293, 365-373.

Hardoim, C.C.P., Costa, R., Araújo, F.V., Hajdu, E., Peixoto, R., Lins, U., et al. (2009). Diversity of bacteria in the marine sponge *Aplysina fulva* in brazilian coastal waters. *Appl. Environ. Microbiol.* 75, 3331-3343. doi:10.1128/AEM.02101-08

Krzanowski, W.J., and Krzanowski, W. (2000). *Principles of multivariate analysis.* (Oxford University Press/Oxford).

Lozupone, C., and Knight, R. (2005). UniFrac: a new phylogenetic method for comparing microbial communities. *Appl. Environ. Microbiol.* 71, 8228-8235. doi:10.1128/AEM.71.12.8228–8235.2005

Manz, W., Amann, R., Ludwig, W., Wagner, M., and Schleifer, K.H. (1992). Phylogenetic oligodeoxynucleotide probes for the major subclasses of *Proteobacteria* - problems and solutions. *Syst. Appl. Microbiol.* 15, 593-600. doi: 10.1016/S0723-2020(11)80121-9

Mclachlan, J. (1964). Some considerations of growth of marine algae in artificial media. *Can. J. Microbiol.* 10, 769-782. doi: 10.1139/m64-098

Meisinger, D.B., Zimmermann, J., Ludwig, W., Schleifer, K.H., Wanner, G., Schmid, M., et al. (2007). *In situ* detection of novel *Acidobacteria* in microbial mats from a chemolithoautotrophically based cave ecosystem (Lower Kane Cave, WY, USA). *Environ. Microbiol.* 9, 1523-1534. doi:10.1111/j.1462-2920.2007.01271.x

Nawrocki, E.P., Kolbe, D.L., and Eddy, S.R. (2009). Infernal 1.0: inference of RNA alignments. *Bioinformatics* 25, 1335-1337. doi:10.1093/bioinformatics/btp157

Neef, A. (1997). *Anwendung der in situ Einzelzell-Identifizierung von Bakterien zur Populationsanalyse in komplexen mikrobiellen Biozönosen.* Technische Universität München.

Quince, C., Lanzen, A., Davenport, R.J., and Turnbaugh, P.J. (2011). Removing noise from pyrosequenced amplicons. *BMC Bioinformatics*. 12, 38. doi:10.1186/1471-2105-12-38.

R Development Core Team (2012) R: A Language and Environment for Statistical Computing. R Foundation for Statistical Computing, Vienna, Austria. Available at: http://www.R-project.org.

Shannon, C.E. (1948a). A mathematical theory of communication. *Bell Syst. Tech. J.* 27, 623-656.

Shannon, C.E. (1948b). A mathematical theory of communication. *Bell Syst. Tech. J.* 27, 379-423.

Smoot, M.E., Ono, K., Ruscheinski, J., Wang, P.L., and Ideker, T. (2011). Cytoscape 2.8: new features for data integration and network visualization. *Bioinformatics* 27, 431-432. doi:10.1093/bioinformatics/btq675

Taylor, J., Schenk, I., Blankenberg, D., and Nekrutenko, A. (2007a). Using Galaxy to perform large-scale interactive data analysis. *Curr. Protoc. Bioinformatic*. doi: 10.1002/0471250953.bi1005s19

Wallner, G., Amann, R., and Beisker, W. (1993). Optimizing fluorescent *in situ* hybridization with ribosomal-RNA-targeted oligonucleotide probes for flow cytometric identification of microorganisms. *Cytometry* 14, 136-143.
