# Supplementary material for: Effects of sample handling and cultivation bias on the specificity of bacterial communities in keratose marine sponges
Source: Front Microbiol. 2014 Nov 18;5:611. doi: 10.3389/fmicb.2014.00611 (PMC4235377; doi:10.3389/fmicb.2014.00611)
Supplement: Supplementary file 1 [file Presentation_1.ZIP › Supplementary Material/Appendix S2.DOCX]

**Appendix S2** PCR-DGGE fingerprinting results and discussion

The PCR-DGGE profiles of *S. spinosulus* obtained with the “direct” and “indirect” cultivation-independent methods were visually very similar, encompassing *c.* eight dominant and several fainter bands consistently found in all samples. Conversely, much larger band variation was observed between *I. variabilis* fingerprints generated with both methods. Profiles obtained for both sponge species via “plate washing” stood in sharp contrast with those generated for cultivation-independent methods, revealing three dominant bands across all samples and a diverse range of fainter bands (**Figure 1A**). Ordination analysis of PCR-DGGE band composition and independent variables clearly discriminated between sample groups (**Figure 1B**). The amount of variation explained by the canonical variables (processing methods and sponge species) was 30.6%. The horizontal axis of the diagram, accounting for 58.8% of the explained variation, differentiated the fingerprints obtained via “direct” and “indirect” cultivation-independent methods from those obtained via “plate washing”. The vertical axis of the diagram grouped all *S. spinosulus* fingerprints generated by both cultivation-independent procedures and *I. variabilis* fingerprints obtained with the “indirect” method apart from *I. variabilis* fingerprints retrieved with the “direct” method. In terms of community structure, Montecarlo permutation tests revealed that the independent variables “*S. spinosulus*” and “direct” and “plate washing” processing methods significantly altered band composition in PCR-DGGE profiles. Conversely, likely due to the large variation in band numbers across profiles, no significant differences in PCR-DGGE richness and diversity measures could be solely attributed to a given sponge species (including all methods) or method (including both sponge species) (*P* > 0.05). Pairwise comparisons of replicates within and between sample groups further revealed statistically similar PCR-DGGE band richness and diversity measures in most cases (**Table S1**).

**Commonalities and discrepancies between PCR-DGGE and 454-pyrosequencing**

Bacterial community profiling and ordination via PCR-DGGE and 454-pyrosequencing were highly congruent in depicting some of the major trends revealed in this study, as follows. First, when quantitative data assessments are applied, the bacterial community associated with *S. spinosulus* differs from that observed in *I. variabilis*, confirming our hypothesis of symbiont specificity in sympatric and closely related sponge species (Hardoim et al., 2012). Second, the use of different cultivation-independent processing methods leads to divergent community structures in *I. variabilis*, but not in *S. spinosulus*. Third, cultivation drastically changes and approximates the shape of otherwise species-specific sponge bacterial communities as determined by cultivation-independent methods. PCR-DGGE failed, nevertheless, at resolving the clear difference in bacterial richness between cultivated and uncultivated sponge bacterial communities, fairly enumerated by 454-pyrosequencing. Altogether, this highlights the usefulness and predictive power of PCR-DGGE fingerprinting and similar techniques in revealing structural shifts between microbial assemblages in their natural settings, even when compared with better resolving next generation sequencing technologies (Cleary et al., 2012). However, much caution is needed when solely using these approaches to catalogue bacterial richness in complex symbiotic communities.

**References**

Cleary, D.F.R., Smalla, K., Mendonça-Hagler, L.C.S., and Gomes, N.C.M. (2012). Assessment of variation in bacterial composition among microhabitats in a mangrove environment using DGGE fingerprints and barcoded pyrosequencing. *Plos One*. 7, e29380. doi:10.1371/journal.pone.0029380

Hardoim, C.C.P., Esteves, A.I.S., Pires, F.R., Gonçalves, J.M.S., Cox, C.J., Xavier, J.R., et al. (2012). Phylogenetically and spatially close marine sponges harbour divergent bacterial communities. *Plos One*. 7, e53029. doi:10.1371/journal.pone.0053029
