# Supplementary material for: Effects of sample handling and cultivation bias on the specificity of bacterial communities in keratose marine sponges
Source: Front Microbiol. 2014 Nov 18;5:611. doi: 10.3389/fmicb.2014.00611 (PMC4235377; doi:10.3389/fmicb.2014.00611)
Supplement: Supplementary file 1 [file Presentation_1.ZIP › Supplementary Material/Appendix S4.DOCX]

**Appendix S4** Taxonomic affiliation of shared and specific OTUs across sponge species and methods

**Full dataset analysis**

The four bacterial OTUs shared by all replicates of *S. spinosulus* under the three processing methods (**Figure 4B**) belonged to *Alphaproteobacteria* (*Rhizobiales* and *Rhodobacterales*), *Gammaproteobacteria* (*Vibrionales*), and *Chloroflexi* (SAR202). For *I. variabilis* the 13 shared OTUs (**Figure 4C**) affiliated with *Acidobacteria* (Sva0725), *Actinobacteria* (*Rubrobacteria*), *Bacteroidetes* (*Flavobacteriia*), *Chloroflexi* (TK17), *Firmicutes* (*Clostridia*), *Poribacteria*, *Alphaproteobacteria* (one *Rhizobiales* OTU and two *Rhodobacterales* OTUs), *Gammaproteobacteria* (one *Alteromonadales* OTU, one *Vibrionales* OTU and two OTUs without order assignment). In *S. spinosulus* (**Figure 4B**), 59 and 44 specific bacterial OTUs were assigned exclusively to the “direct” [OTUs classified as *Firmicutes* (*Clostridiales*) and *Betaproteobacteria* (*Burkholderiales*) as the most abundant] and “indirect” [OTUs classified as *Poribacteria* and *Alphaproteobacteria* (*Rhodobacterales*) as the most abundant] methods, respectively. For *I. variabilis* (**Figure 4C**) 93 OTUs specific to the “direct” method were found, with OTUs in the *Poribacteria*, *Gammaproteobacteria* and *Deltaproteobacteria* (*Syntrophobacterales*) groups as the most representative, whereas 96 OTUs exclusive to the “indirect” method were observed, of which OTUs belonging to *Acidobacteria* (Sva0725), *Chloroflexi* (*Anaerolineae*) and *Planctomycetes* (*Planctomycetia*) were the most abundant. *S. spinosulus* contained 25 exclusive bacterial OTUs obtained with the “plate washing” method (**Figure 4B**), of which *Gammaproteobacteria* in the orders *Alteromonadales* and *Vibrionales* were the most abundant. From the same method, *I. variabilis* harboured 15 specific bacterial OTUs (**Figure 4C**) among which *Gammaproteobacteria* (*Alteromonadales*) and *Alphaproteobacteria* (*Kiloniellales*) were the most abundant.

Among the 167 bacterial OTUs common to *S. spinosulus* and *I. variabilis* across all methods (**Figure 4D**), *Alphaproteobacteria* (*Rhizobiales*), *Acidobacteria* (Sva0725), *Actinobacteria* (*Acidimicrobiia*) and *Poribacteria* clades were the most abundant. The four common OTUs found between *S. spinosulus* under all methods and seawater (**Figure 4E**) were classified as *Alphaproteobacteria* (*Rhizobiales* and *Rhodobacterales*), *Gammaproteobacteria* (*Vibrionales*) and *Chloroflexi* (SAR202). The five bacterial OTUs shared between *I. variabilis* and seawater (**Figure 4F**) affiliated with *Alphaproteobacteria* (*Rhizobiales*), *Acidobacteria* (Sva0275), *Gammaproteobacteria* (*Vibrionales*), *Actinobacteria* (*Rubrobacteria*) and *Firmicutes* (*Clostridia*). From the 32 bacterial OTUs shared by both sponge species processed via both cultivation-independent methods and seawater (**Figure 4G**), *Acidobacteria* (Sva0725), *Actinobacteria* (*Acidimicrobiia*), *Poribacteria* and PAUC34f were the most abundant, even though they were very rare in seawater (< 10 sequences in each OTU).

**Analysis of dominant OTUs (rare bacteriome removed)**

When only OTUs with at least 50 sequences were analysed (**Figure S3A-G**), the same four OTUs [*Alphaproteobacteria* (*Rhizobiales* and *Rhodobacterales*), *Gammaproteobacteria* (*Vibrionales*) and *Chloroflexi* (SAR202)] were found in *S. spinosulus* under the three processing methods (**Figure S3B**). However, for *I. variabilis* the number of common OTUs dropped from 13 to nine (**Figure S3C**). These belonged to *Acidobacteria* (Sva0725), *Actinobacteria* (*Rubrobacteria*), *Bacteroidetes* (*Flavobacteriia*), *Chloroflexi* (TK17), *Poribacteria*, *Alphaproteobacteria* (*Rhizobiales*) and *Gammaproteobacteria* (*Alteromonadales*, *Vibrionales* and one OTU without order assignment). For both sponge species, the number of so-called “method-specific” (**Figure S3B,C**) and “species-specific” (**Figure S3D**) OTUs sharply decreased when compared with the full dataset analysis (**Figure 4B-D**). Further, independently of the processing methods, both sponge species shared 90 “dominant” OTUs (**Figure S3D**), of which the most profuse were assigned to *Alphaproteobacteria* (*Rhizobiales*), *Acidobacteria* (Sva0725) and *Actinobacteria* (*Acidimicrobiia*) (**Figure S3D**).

After removal of the rare OTUs, still four OTUs were common to *S. spinosulus* under the three processing methods and seawater (**Figure S3E**). These OTUs represented exactly the same phyla found in the full data set analysis (**Figure 4E**), and affiliated with *Alphaproteobacteria* (*Rhizobiales* and *Rhodobacterales*), *Gammaproteobacteria* (*Vibrionales*) and *Chloroflexi* (SAR202). *I. variabilis* and seawater shared four OTUs that belonged to *Acidobacteria* (Sva0725), *Actinobacteria* (*Rubrobacteria*), *Alphaproteobacteria* (*Rhizobiales*) and *Gammaproteobacteria* (*Vibrionales*) (**Figure S3F**). Finally, both sponge species under culture-independent methods and seawater contained six common OTUs affiliated with *Actinobacteria* (*Acidimicrobiia* and *Rubrobacteria*), *Acidobacteria* (Sva0725), *Chloroflexi* (SAR202) and *Alphaproteobacteria* (*Rhizobiales*) (**Figure S3G**).

Regarding the “rare” symbionts as a whole, that is, the pool of OTUs containing less than 50 sequences through the whole dataset, we noticed that they represented a rather diverse community. In *S. spinosulus*, 163 such OTUs were found. Among them, 86 OTUs were specific to this sponge species. For *I. variabilis*, from 236 rare OTUs 160 were exclusive to this sponge species. Independently of the sponge species, these specific OTUs encompassed sponge-associated phyla commonly enriched in marine sponges such as *Acidobacteria*, *Actinobacteria*, *Chloroflexi*, *Proteobacteria* (*Alpha* and *Gamma* classes) and *Poribacteria* (**Table S2A,B**).
