# Supplementary material for: Effects of sample handling and cultivation bias on the specificity of bacterial communities in keratose marine sponges
Source: Front Microbiol. 2014 Nov 18;5:611. doi: 10.3389/fmicb.2014.00611 (PMC4235377; doi:10.3389/fmicb.2014.00611)
Supplement: Supplementary file 1 [file Presentation_1.ZIP › Supplementary Material/Appendix S7.DOCX]

**Appendix S7** Distribution of *Poribacteria* OTUs across sample categories

In total, ten OTUs (97% sequence similarity) were assigned to the candidate phylum *Poribacteria*. They were recovered from six of the seven sample categories. The only sample category in which a poribacterial hit was not found was *S. spinosulus* treated with the “plate washing” processing method (**Table App7-1**).

**Table App7-1** Distribution of *Poribacteria* OTUs in all libraries

| **OTU_IDs** | **Ss_D***^1^* | **Ss_I** | **Ss_PW** | **Iv_D** | **Iv_I** | **Iv_PW** | **SW** |
| --- | --- | --- | --- | --- | --- | --- | --- |
| **127** | 0*^2^* | 0 | 0 | 0 | 0 | 0 | 8 |
| **207** | 0 | 0 | 0 | 8 | 6 | 0 | 0 |
| **263** | 1 | 1 | 0 | 0 | 0 | 0 | 0 |
| **473** | 0 | 34 | 0 | 32 | 2 | 1 | 0 |
| **492** | 2 | 0 | 0 | 0 | 0 | 0 | 0 |
| **678** | 171 | 159 | 0 | 58 | 0 | 0 | 0 |
| **768** | 3512 | 1940 | 0 | 219 | 2 | 0 | 9 |
| **1593** | 2 | 0 | 0 | 0 | 0 | 0 | 0 |
| **2081** | 0 | 0 | 0 | 0 | 4 | 0 | 0 |
| **2175** | 38 | 3 | 0 | 1 | 0 | 0 | 0 |
| **Total** | 3726 | 2137 | 0 | 318 | 14 | 1 | 17 |
| **Abundance*^3^*** | 59.97% | 34.39% | 0% | 5.11% | 0.22% | 0.016% | 0.27% |

^a^Ss_D, Ss_I and Ss_PW: *S. spinosulus* with “direct”, “indirect” and “plate washing” processing methods. Iv_D, Iv_I, IV_PW: *I. variabilis* with “direct”, “indirect” and “plate washing” processing methods. SW: seawater.

^b^Number of sequences recovered in each sample per OTU.

^c^Relative abundance of poribacterial sequences in each sample category.

A comprehensive phylogenetic inference of poribacterial sequences retrieved in this study and representative sequences collected worldwide from several sponge hosts and oceans was performed as described by Hardoim et al. (2013). Briefly, representative sequences of each OTU found in this study were aligned using the SINA web aligner (Pruesse et al., 2007) and imported into a SILVA 16S rRNA database version 102 containing pre-aligned poribacterial sequences of diverse origins (Hardoim et al., 2013) using the parsimony tool as implemented in the ARB software (Ludwig et al., 2004). The 16S rRNA gene sequences of type strains and uncultured representatives of *Poribacteria*-related phyla (Fieseler et al., 2004; Wagner and Horn, 2006) were included in the alignment procedure, and final alignments were manually checked. Poorly-aligned sites were identified and excluded from further analysis using PAUP* (vers. 4.0b10; Swofford, 2003). The general-time reversible model (GTR; Rodriguez et al., 1990) with a discrete gamma-distribution of among-site rate variation (Γ_4_) and a proportion of invariant sites (I) was the most suitable evolutionary model for phylogenetic reconstruction as determined using MrModeltest (vers. 2.3; Nylander 2008). Phylogenetic inference was performed by Maximum Likelihood using RAxML (vers. 7.0.4-MPI; Stamatakis, 2006) as explained by Hardoim et al. (2013). The resulting phylogenetic tree can be seen in **Figure** **AppS7-1** (below). In general, the phylum *Poribacteria* was more abundant in *S. spinosulus* than in *I. variabilis*. A sharp decrease in the representativeness of poribacterial hits was observed in *I. variabilis* handled with the “indirect” method in comparison with the “direct” method, whereas such an effect was negligible in *S. spinosulus*. The most abundant poribacterial phylotype in both sponge species (OTU 768) resembled, along with other less dominant OTUs, poribacterial symbionts of *Aplysina aerophoba* from the Mediterranean Sea (**Figure** **AppS7-1**). Unexpectedly, beyond its detection in both sponge species by cultivation independent methods, OTU 473 was also recovered from *I. variabilis* via plate washing, being represented by one single sequence in this sample group. This OTU formed a supported phylogenetic cluster with poribacterial sequences from the marine sponges *Aplysina* *fulva* and *Plakortis* sp. collected in Brazil and Bahamas, respectively (**Figure** **AppS7-1**). This outcome possibly constitutes the first testimony to the cultivability of the *Poribacteria* - but **not** of their isolation -, and as such deserves careful consideration and cautious interpretation.

Cultivation-independent genomics of *Poribacteria* revealed aerobic and heterotrophic metabolism capacities with major parts of the glycolysis, tricarboxylic acid cycle and pentose phosphate pathways being observed (Siegl et al., 2011). The marine agar medium contains, beyond many minerals, peptone and yeast extract that may be used as carbon (glucose and amino acids) and energy source for poribacterial growth. However, this sponge-enriched bacterium has been resistant to all cultivation efforts performed so far. Based on the documentation of *Poribacteria*, *Acidobacteria* and *Chloroflexi* on Marine Agar plates reported in this study, chemical cues obtained from the host (*e.g.* sponge-derived extracts), often assumed as a pre-requisite for the growth of “true” microbial mutualists, may not be strictly necessary to retrieve some of the typical sponge symbionts in culture. Nevertheless, the use of sponge-derived extracts certainly remains an reasonable alternative to enhance cultivation efficiencies since we believe that, owing to the detection of the abovementioned bacteria in extremely low numbers, marine agar culturing is clearly not conducive to the isolation of the most typical (or abundant) bacterial symbionts of marine sponges. The unexpected registration of *Poribacteria*, *Chloroflexi* and *Acidobacteria* OTUs on our culture plates may result from overcame hurdles of the traditional colony picking-and-isolation method in fully disentangling the diversity of culturable bacterial communities. Typically, this procedure routinely discards microorganisms that grow in syntrophy given the *a priori* necessity to obtain pure cultures. Moreover, it often disregards so-called micro-colonies that escape observation by the naked eye, one of the major reasons why the *Acidobacteria*, for instance, remained uncultivated for several years (Davis et al., 2011). Further, colony picking-and-isolation methods are restricted by sampling effort, a limitation that we were able to bypass via “plate washing” coupled to high throughput sequencing of the mixed cultured community, resulting in full diversity coverage of the cultivated sponge-associated microbiome. Although our strategy likely enabled superior detection power than regular sampling procedures, it did not enable the isolation of the detected cultures. Future efforts in captivating marine sponge symbionts will probably benefit from multifaceted approaches whereby alternative medium formulation, inoculation and incubation conditions are combined with innovative methods of data acquisition and analysis.


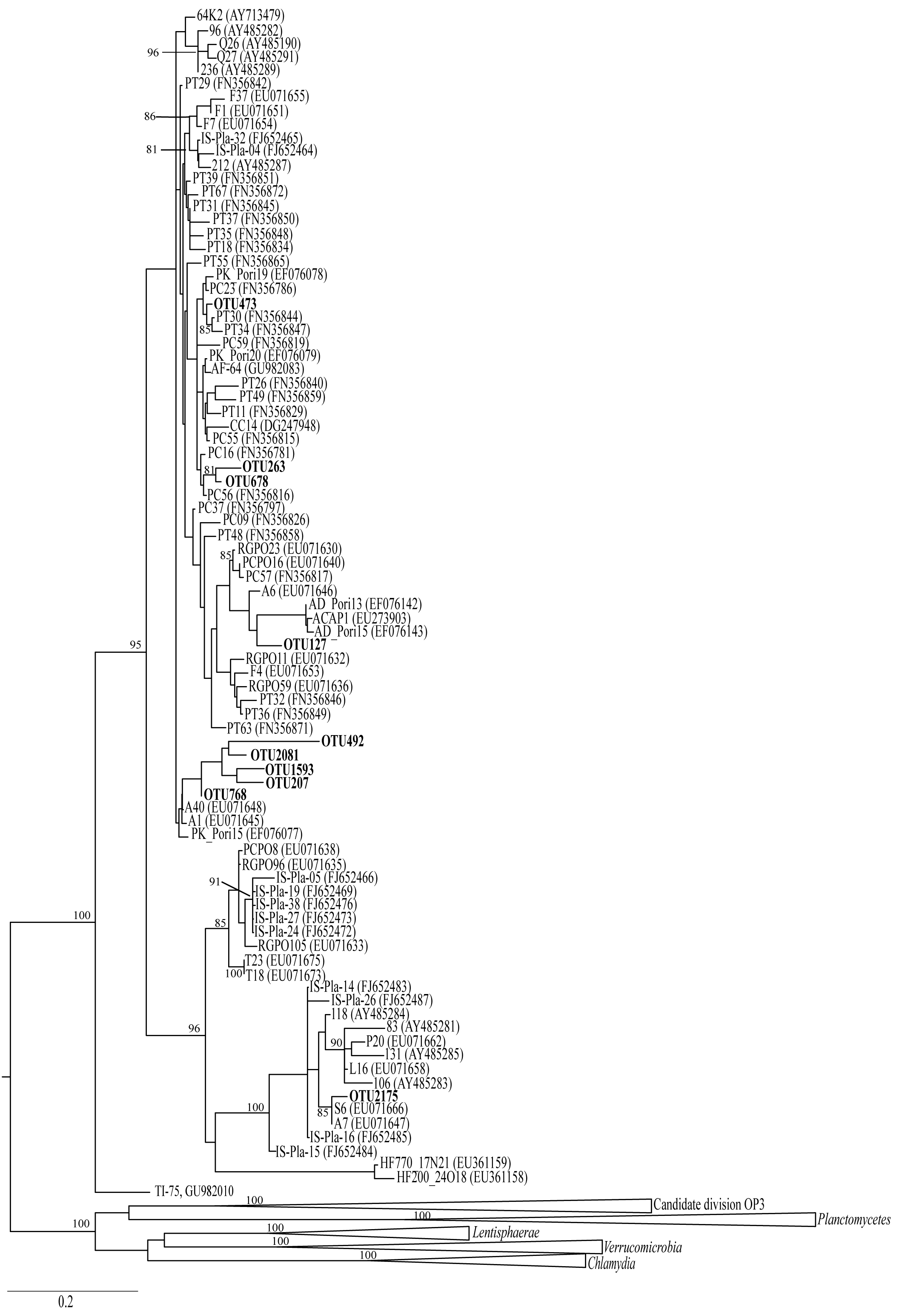


**Figure AppS7-1**. Phylogenetic inference of poribacterial 16S rRNA gene sequences. The optimal Maximum Likelihood tree is shown, with poribacterial OTUs identified in this study highlighted in bold. Hardoim et al. (2013) have recently inferred the phylogenetic relationships of all other poribacterial entries. Numbers at tree nodes are bootstrap values calculated by Maximum Likelihood, and values above 75 are shown.

**References**

Davis, K.E.R., Sangwan, P., and Janssen, P.H. (2011). *Acidobacteria*, *Rubrobacteridae* and *Chloroflexi* are abundant among very slow-growing and mini-colony-forming soil bacteria. *Environ. Microbiol.* 13, 798-805. doi:10.1111/j.1462-2920.2010.02384.x

Fieseler, L., Horn, M., Wagner, M., and Hentschel, U. (2004). Discovery of the novel candidate phylum "*Poribacteria*" in marine sponges. *Appl. Environ. Microbiol.* 70, 3724-3732. doi: 10.1128/AEM.70.6.3724–3732.2004

Hardoim, C.C.P., Cox, C.J., Peixoto, R.S., Rosado, A.S., Costa, R., and van Elsas, J.D. (2013). Diversity of the candidate phylum *Poribacteria* in the marine sponge *Aplysina fulva*. *Braz. J. Microbiol.* 44, 329-334. doi: 10.1590/S1517-83822013000100048

Ludwig, W., Strunk, O., Westram, R., Richter, L., Meier, H., Yadhukumar, A.B., et al (2004). ARB: a software environment for sequence data. *Nucleic Acids Res.* 32, 1363-1371. doi: 10.1093/nar/gkh293

Nylander, J.A.A. (2008). MrModeltest ver. 2.3. Available at: http://www.abc.se/~nylander/mrmodeltest2/mrmodeltest2.html.

Pruesse, E., Quast, C., Knittel, K., Fuchs, B.M., Ludwig, W.G., Peplies, J., et al (2007). SILVA: a comprehensive online resource for quality checked and aligned ribosomal RNA sequence data compatible with ARB. *Nucleic Acids Res.* 35, 7188-7196. doi:10.1093/nar/gkm864

Rodriguez, F., Oliver, J.L., Marin, A., and Medina, J.R. (1990). The general stochastic-model of nucleotide substitution. *J. Theor. Biol.* 142, 485-501.

Siegl, A., Kamke, J., Hochmuth, T., Piel, J., Richter, M., Liang, C.G., et al. (2011). Single-cell genomics reveals the lifestyle of *Poribacteria*, a candidate phylum symbiotically associated with marine sponges. *ISME J.* 5, 61-70. doi:10.1038/ismej.2010.95

Stamatakis, A. (2006). RAxML-VI-HPC: Maximum likelihood-based phylogenetic analyses with thousands of taxa and mixed models. *Bioinformatics* 22, 2688-2690.

Swofford D.L. (2003). PAUP. Phylogenetic Analysis Using Parsimony (and Other Methods), ver. 4. (Sinauer Associates, Sunderland). Available at: http://paup.csit.fsu.edu/downl.html.

Wagner, M., and Horn, M. (2006). The *Planctomycetes*, *Verrucomicrobia*, *Chlamydiae* and sister phyla comprise a superphylum with biotechnological and medical relevance. *Curr. Opin. Biotech.* 17, 241-249. doi: 10.1016/j.copbio.2006.05.005
