# Supplementary material for: Effects of sample handling and cultivation bias on the specificity of bacterial communities in keratose marine sponges
Source: Front Microbiol. 2014 Nov 18;5:611. doi: 10.3389/fmicb.2014.00611 (PMC4235377; doi:10.3389/fmicb.2014.00611)
Supplement: Supplementary file 1 [file Presentation_1.ZIP › Supplementary Material/Table S1.DOCX]

**Table S1** Statistical analysis of PCR-DGGE band richness and diversity

1. richness measures, factorial ANOVA

|  | *Degrees of Freedom* | | *Sum of squares* | | *Mean of squares* | | *F value* | | *Pr(>F)* | |  |
| --- | --- | --- | --- | --- | --- | --- | --- | --- | --- | --- | --- |
| Species | 1 | | 105.5 | | 105.5 | | 1.041 | | 0.322 | |  |
| Method | 2 | | 291.1 | | 145.5 | | 1.436 | | 0.265 | |  |
| Species:Method | 2 | | 1060.4 | | 530.2 | | 5.232 | | **0.017** | |  |
| Residuals | 17 | | 1722.9 | | 101.3 | |  | |  | |  |
|  | |  | |  | |  | |  | |  |  |
| (b) richness measures, TukeyHSD | | | | | | | | | | | |
| *Species* | | *difference* | | *lower* | | *upper* | | *p adj* | |  | |
| Ss-Iv | | 4.287879 | | -4.578149 | | 13.15391 | | 0.3218551 | |  | |
|  | |  | |  | |  | |  | |  | |
| *Method* | | *difference* | | *lower* | | *upper* | | *p adj* | |  | |
| I-D | | 1.341991 | | -12.024182 | | 14.70817 | | 0.9641663 | |  | |
| PW-D | | 8.091991 | | -5.274182 | | 21.45817 | | 0.2922774 | |  | |
| PW-I | | 6.75 | | -6.162951 | | 19.66295 | | 0.3928006 | |  | |
|  | |  | |  | |  | |  | |  | |
| *Species:Method* | | *difference* | | *lower* | | *upper* | | *p adj* | |  | |
| Ss_D-Iv_D | | 19.916667 | | -4.677965 | | 44.511298 | | 0.15321 | |  | |
| Iv_I-Iv_D | | 7.916667 | | -16.677965 | | 32.511298 | | 0.9014505 | |  | |
| Ss_I-Iv_D | | 16.916667 | | -7.677965 | | 41.511298 | | 0.2873625 | |  | |
| Iv_PW-Iv_D | | 25.666667 | | 1.072035 | | 50.261298 | | **0.0380796** | |  | |
| Ss_PW-Iv_D | | 12.666667 | | -11.927965 | | 37.261298 | | 0.5810021 | |  | |
| Iv_I-Ss_D | | -12 | | -34.770204 | | 10.770204 | | 0.5581326 | |  | |
| Ss_I-Ss_D | | -3 | | -25.770204 | | 19.770204 | | 0.9979853 | |  | |
| Iv_PW-Ss_D | | 5.75 | | -17.020204 | | 28.520204 | | 0.962147 | |  | |
| Ss_PW-Ss_D | | -7.25 | | -30.020204 | | 15.520204 | | 0.905361 | |  | |
| Ss_I-Iv_I | | 9 | | -13.770204 | | 31.770204 | | 0.799518 | |  | |
| Iv_PW-Iv_I | | 17.75 | | -5.020204 | | 40.520204 | | 0.1805367 | |  | |
| Ss_PW-Iv_I | | 4.75 | | -18.020204 | | 27.520204 | | 0.9833062 | |  | |
| Iv_PW-Ss_I | | 8.75 | | -14.020204 | | 31.520204 | | 0.8169555 | |  | |
| Ss_PW-Ss_I | | -4.25 | | -27.020204 | | 18.520204 | | 0.9898365 | |  | |
| Ss_PW-Iv_PW | | -13 | | -35.770204 | | 9.770204 | | 0.4760904 | |  | |

(c) diversity indices, factorial ANOVA

|  | *Degrees of Freedom* | | | *Sum of squares* | | | | *Mean of squares* | | | | *F value* | | | | *Pr(>F)* | | | | |  |  |  |
| --- | --- | --- | --- | --- | --- | --- | --- | --- | --- | --- | --- | --- | --- | --- | --- | --- | --- | --- | --- | --- | --- | --- | --- |
| Species | 1 | | | 0.1064 | | | | 0.1064 | | | | 2.282 | | | | 0.1493 | | | | |  |  |  |
| Method | 2 | | | 0.0358 | | | | 0.0179 | | | | 0.384 | | | | 0.6869 | | | | |  |  |  |
| Species:Method | 2 | | | 0.7167 | | | | 0.3584 | | | | 7.683 | | | | **0.0042** | | | | |  |  |  |
| Residuals | 17 | | | 0.793 | | | | 0.0466 | | | |  | | | |  | | | | |  |  |  |
| (d) diversity indices, TukeyHSD | | | | | | | | | | | | | | | | |  |  |  |  |  |  |  |
| *Species* | | | *difference* | | *lower* | | | | *upper* | | | | *p adj* | | | |  | | | | |  |  |
| Ss-Iv | | | 0.1361803 | | -0.0540256 | | | | 0.3263863 | | | | 0.1492673 | | | |  | | | | |  |  |
|  | | |  | |  | | | |  | | | |  | | | |  | | | | |  |  |
| *Method* | | | *difference* | | *lower* | | | | *upper* | | | | *p adj* | | | | |  | | | | |  |
| I-D | | | 0.05516365 | | -0.2315855 | | | | 0.3419128 | | | | 0.8753295 | | | | |  | | | | |  |
| PW-D | | | 0.09771765 | | -0.1890315 | | | | 0.3844668 | | | | 0.6632777 | | | | |  | | | | |  |
| PW-I | | | 0.042554 | | -0.234472 | | | | 0.31958 | | | | 0.9183697 | | | | |  | | | | |  |
|  | | |  | |  | | | |  | | | |  | | | |  | | | | |  |  |
| *Species:Method* | | *difference* | | | | *lower* | | | | *upper* | | | | *p adj* | | | | |  |  |  |  |  |
| Ss_D-Iv_D | | 0.5325888 | | | | | 0.004951765 | | | | 1.0602259 | | | | **0.0471701** | | | | |  | | | |
| Iv_I-Iv_D | | 0.2173451 | | | | | -0.310291985 | | | | 0.7449822 | | | | 0.7718312 | | | | |  | | | |
| Ss_I-Iv_D | | 0.4822008 | | | | | -0.045436235 | | | | 1.0098379 | | | | 0.0843655 | | | | |  | | | |
| Iv_PW-Iv_D | | 0.5518168 | | | | | 0.024179765 | | | | 1.0794539 | | | | **0.0375465** | | | | |  | | | |
| Ss_PW-Iv_D | | 0.2328371 | | | | | -0.294799985 | | | | 0.7604742 | | | | 0.7200901 | | | | |  | | | |
| Iv_I-Ss_D | | -0.3152437 | | | | | -0.803740753 | | | | 0.1732533 | | | | 0.3496245 | | | | |  | | | |
| Ss_I-Ss_D | | -0.050388 | | | | | -0.538885003 | | | | 0.438109 | | | | 0.9993781 | | | | |  | | | |
| Iv_PW-Ss_D | | 0.019228 | | | | | -0.469269003 | | | | 0.507725 | | | | 0.9999946 | | | | |  | | | |
| Ss_PW-Ss_D | | -0.2997517 | | | | | -0.788248753 | | | | 0.1887453 | | | | 0.4010778 | | | | |  | | | |
| Ss_I-Iv_I | | 0.2648558 | | | | | -0.223641253 | | | | 0.7533528 | | | | 0.5293855 | | | | |  | | | |
| Iv_PW-Iv_I | | 0.3344718 | | | | | -0.154025253 | | | | 0.8229688 | | | | 0.2916672 | | | | |  | | | |
| Ss_PW-Iv_I | | 0.015492 | | | | | -0.473005003 | | | | 0.503989 | | | | 0.9999982 | | | | |  | | | |
| Iv_PW-Ss_I | | 0.069616 | | | | | -0.418881003 | | | | 0.558113 | | | | 0.997079 | | | | |  | | | |
| Ss_PW-Ss_I | | -0.2493638 | | | | | -0.737860753 | | | | 0.2391333 | | | | 0.5896977 | | | | |  | | | |
| Ss_PW-Iv_PW | | -0.3189798 | | | | | -0.807476753 | | | | 0.1695173 | | | | 0.337834 | | | | |  | | | |

Ss, *Sarcotragus spinosulus*; Iv, *Ircinia variabilis*; D, direct processing method; I, indirect processing method; PW, plate washing processing method.
