# Supplementary figures and images for: Effects of sample handling and cultivation bias on the specificity of bacterial communities in keratose marine sponges
Source: Front Microbiol. 2014 Nov 18;5:611. doi: 10.3389/fmicb.2014.00611 (PMC4235377; doi:10.3389/fmicb.2014.00611)

(A) Phylum-level

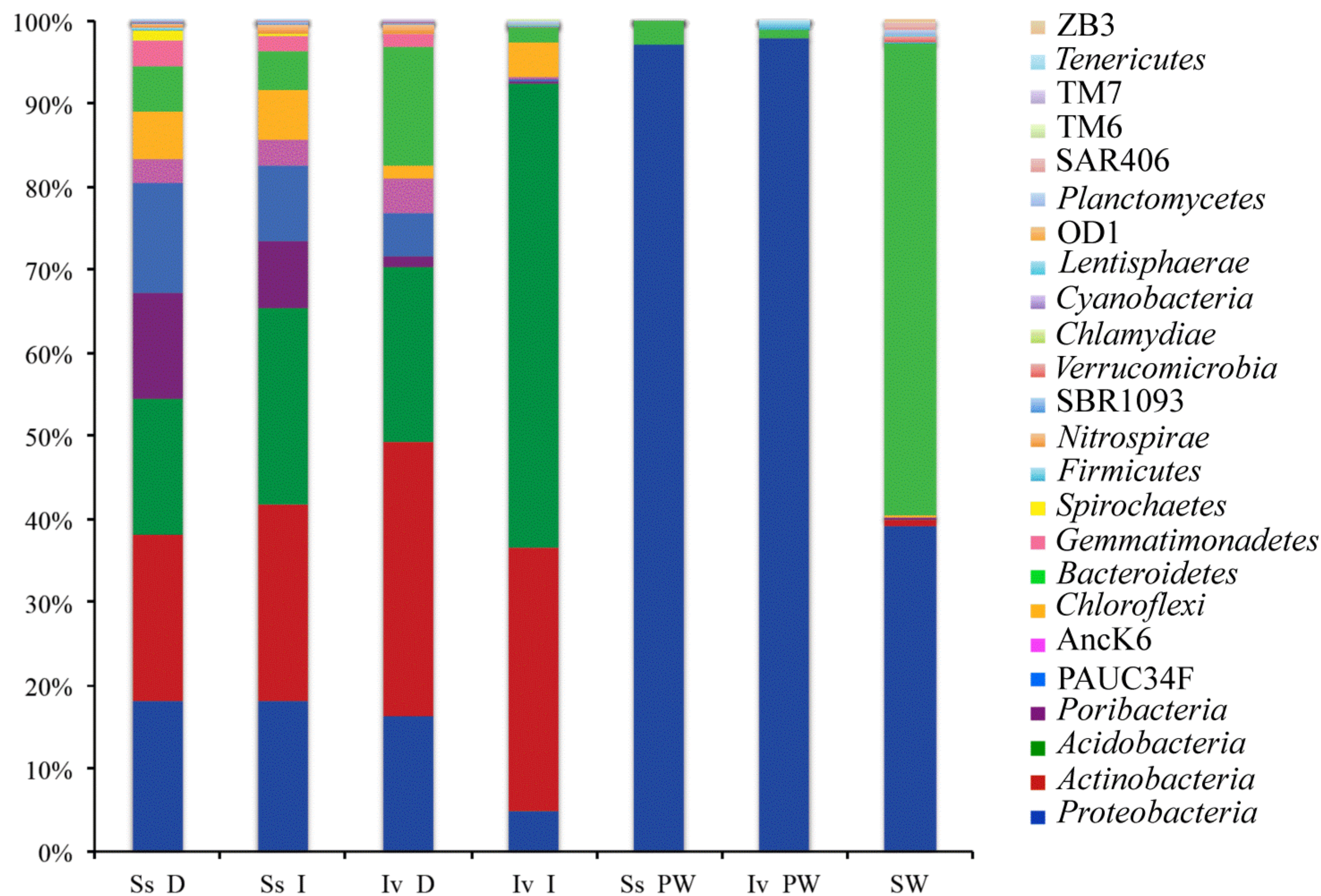

Appendix S3

(B) Class-level

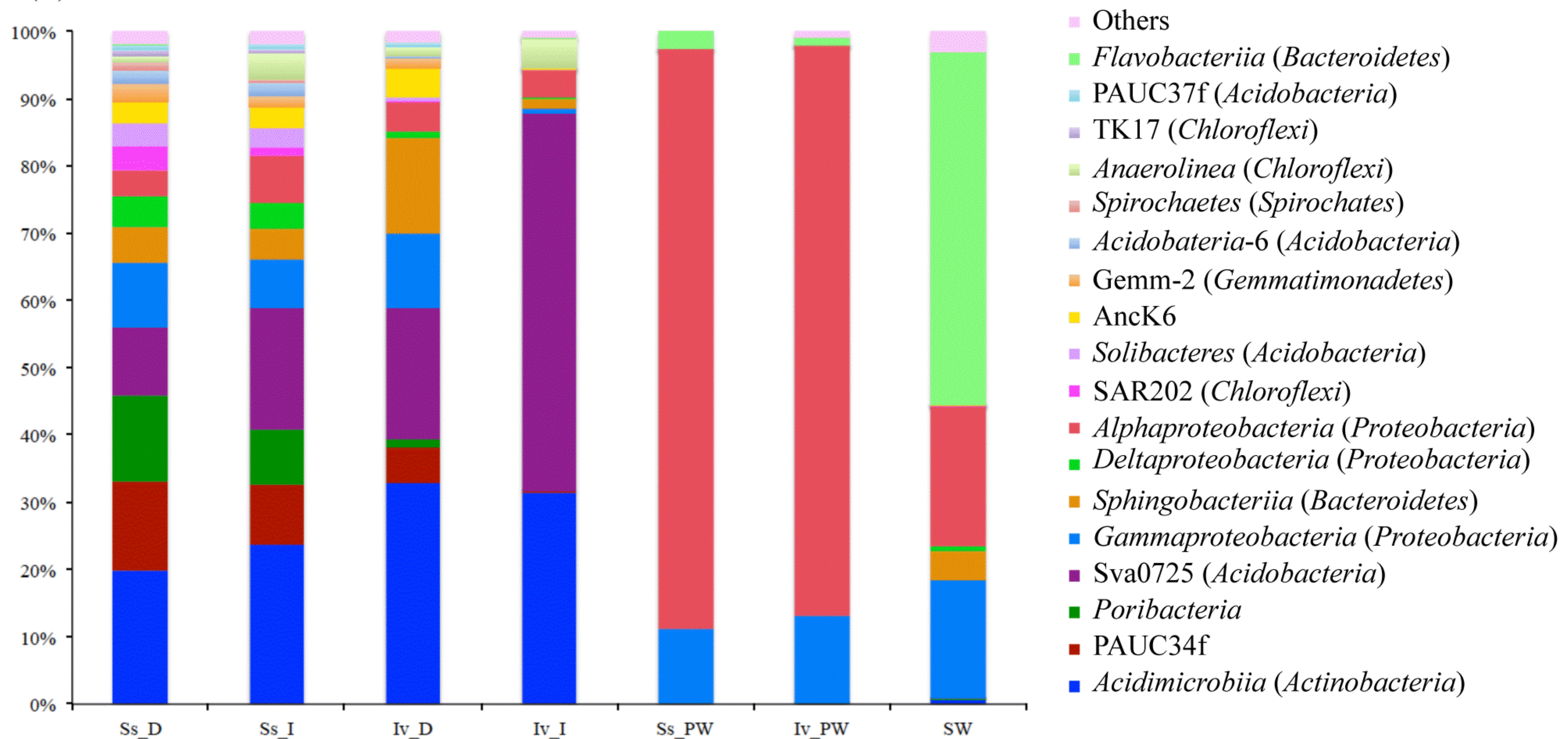

Supplement: Supplementary file 1 [file Presentation_1.ZIP › Supplementary Material/Appendix S3.PDF]

(A)

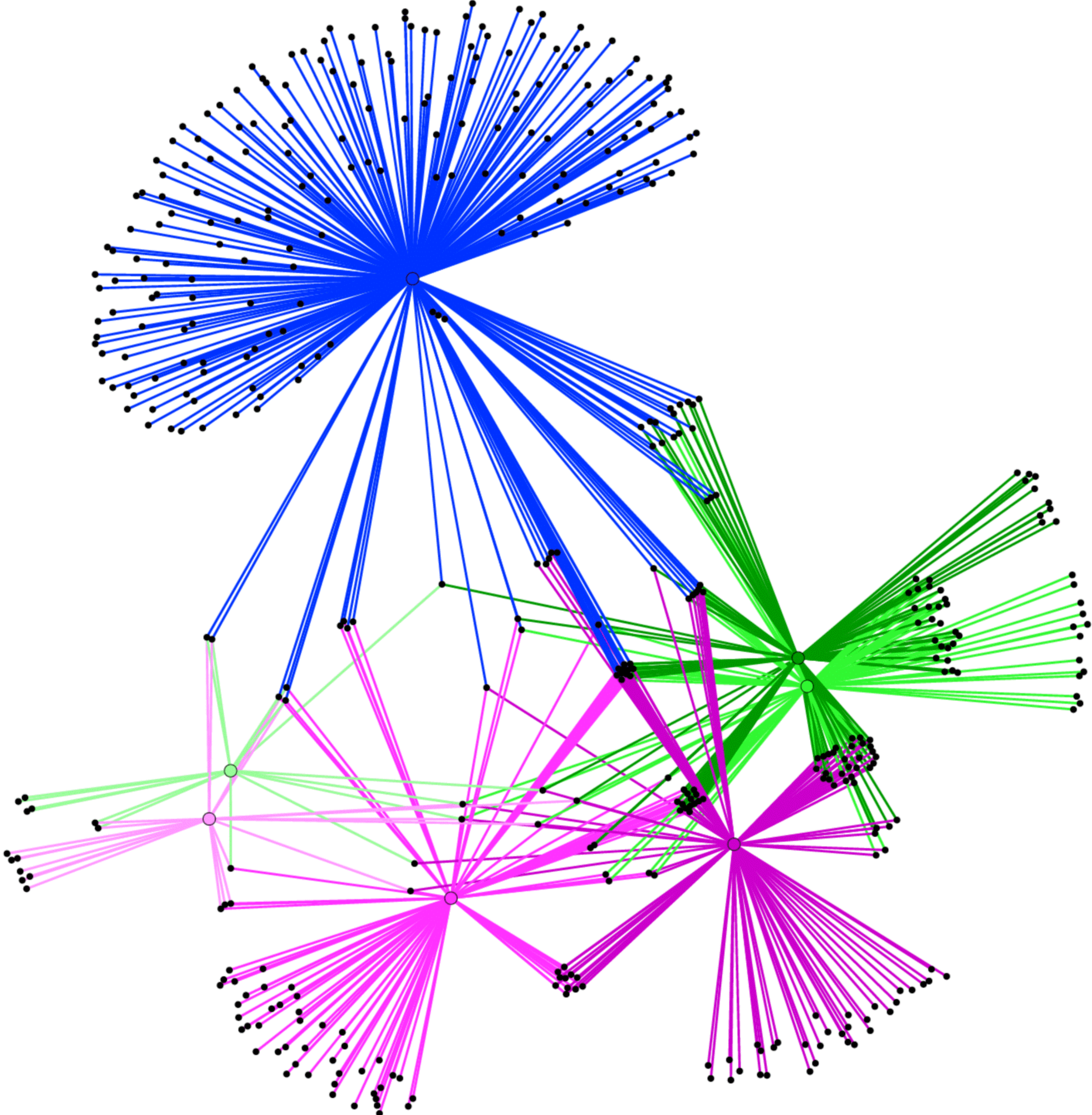

(B)

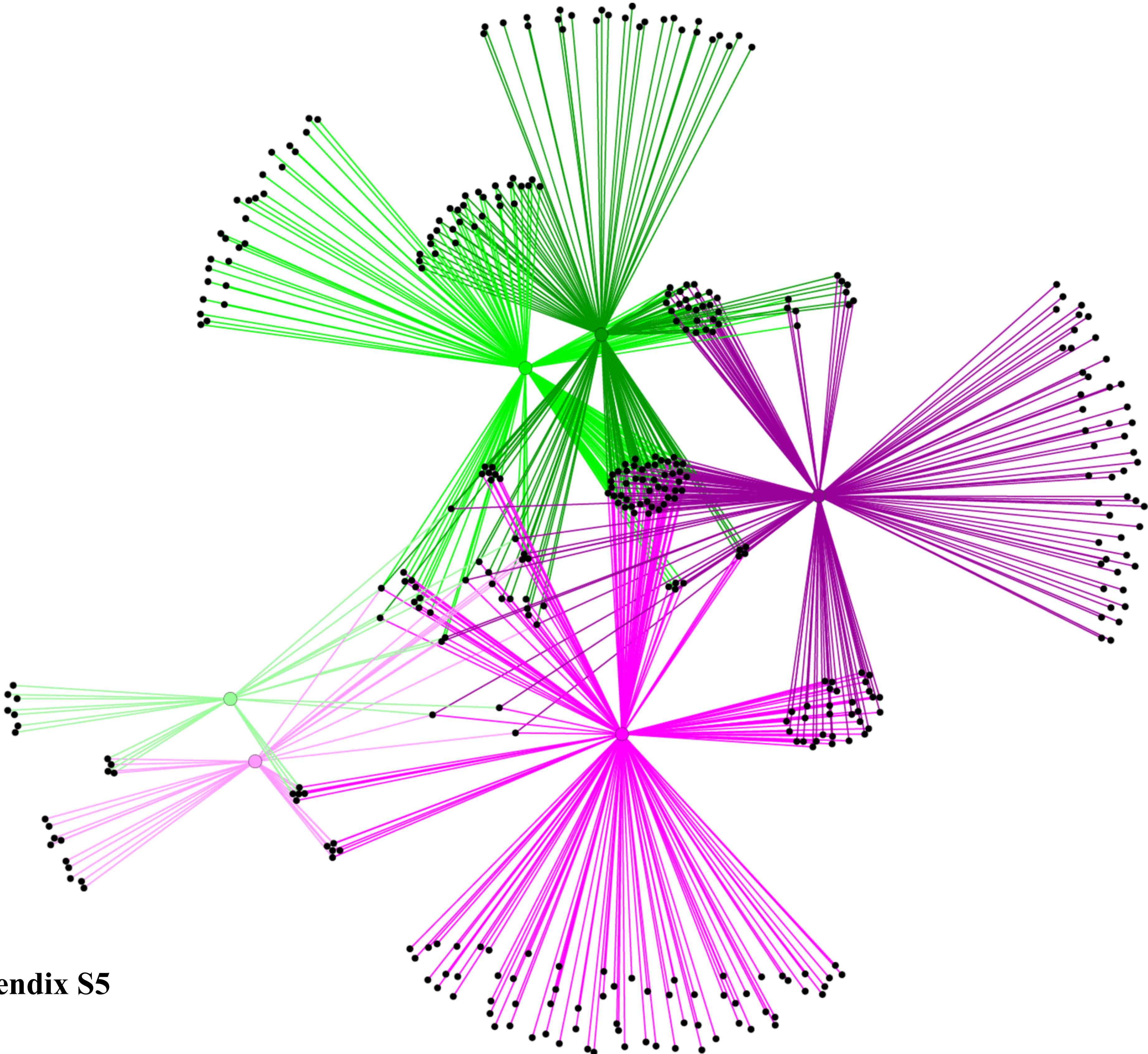

Supplement: Supplementary file 1 [file Presentation_1.ZIP › Supplementary Material/Appendix S5.PDF]

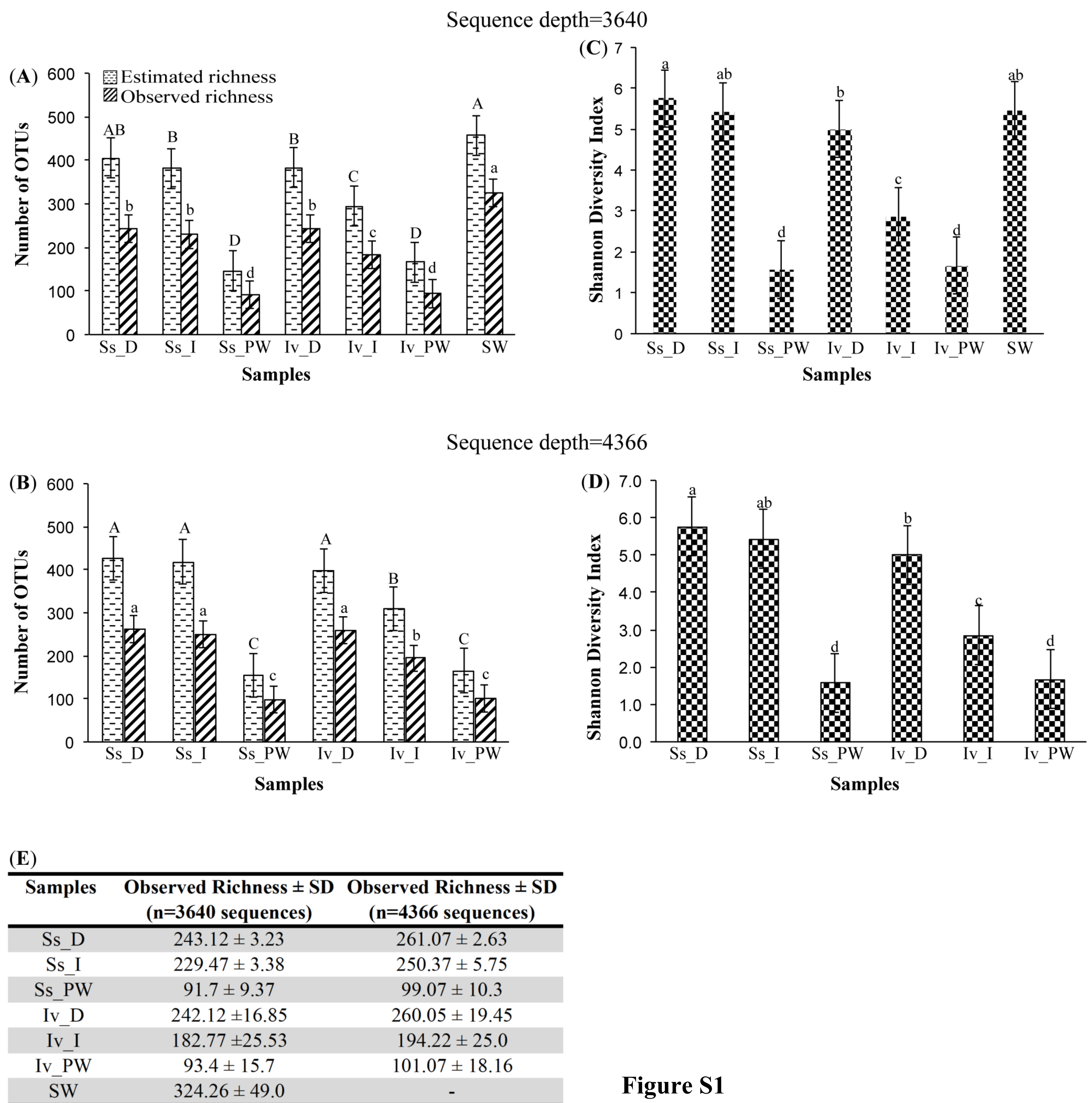

Figure S1

Supplement: Supplementary file 1 [file Presentation_1.ZIP › Supplementary Material/Figure S1.PDF]

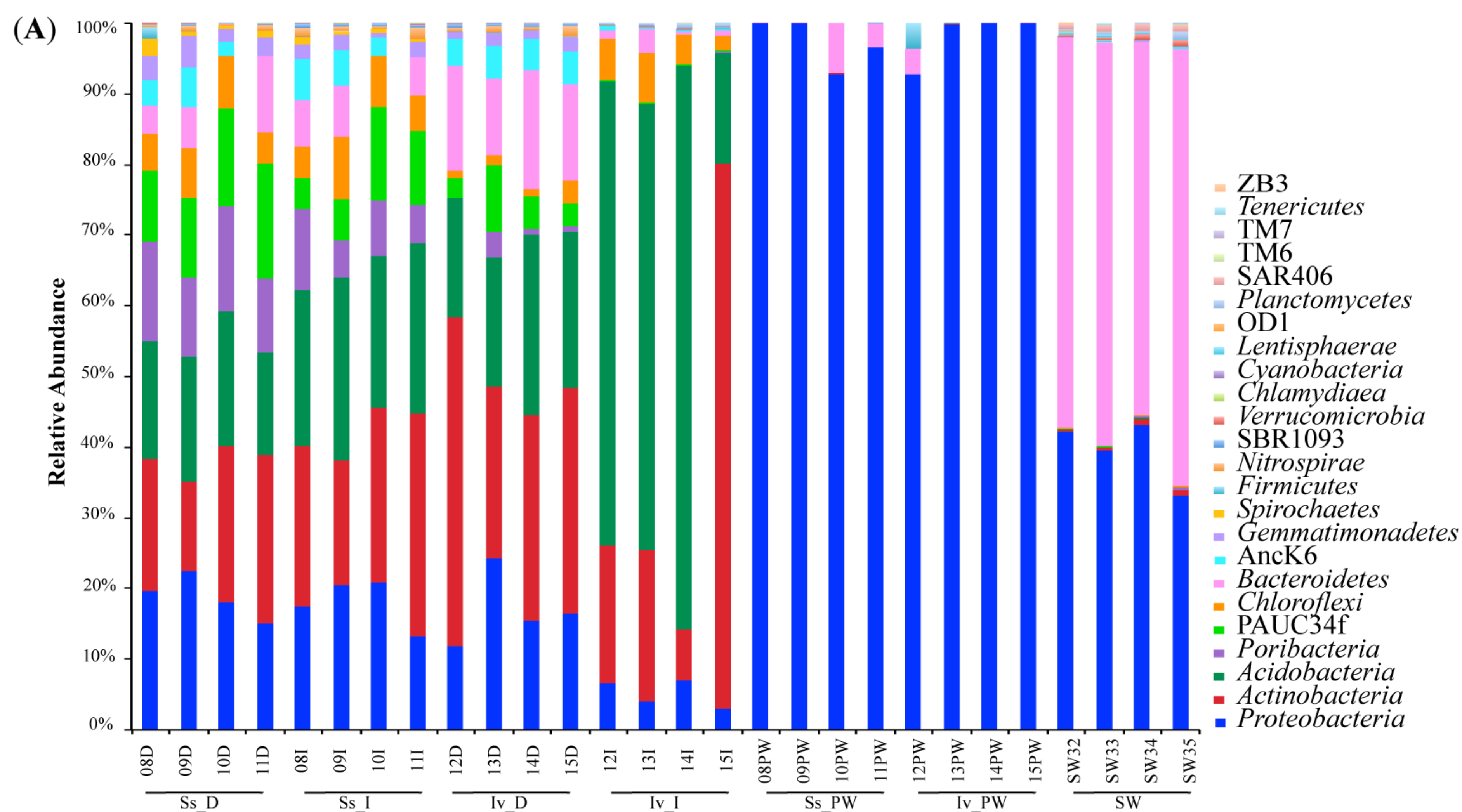

**Figure S2**

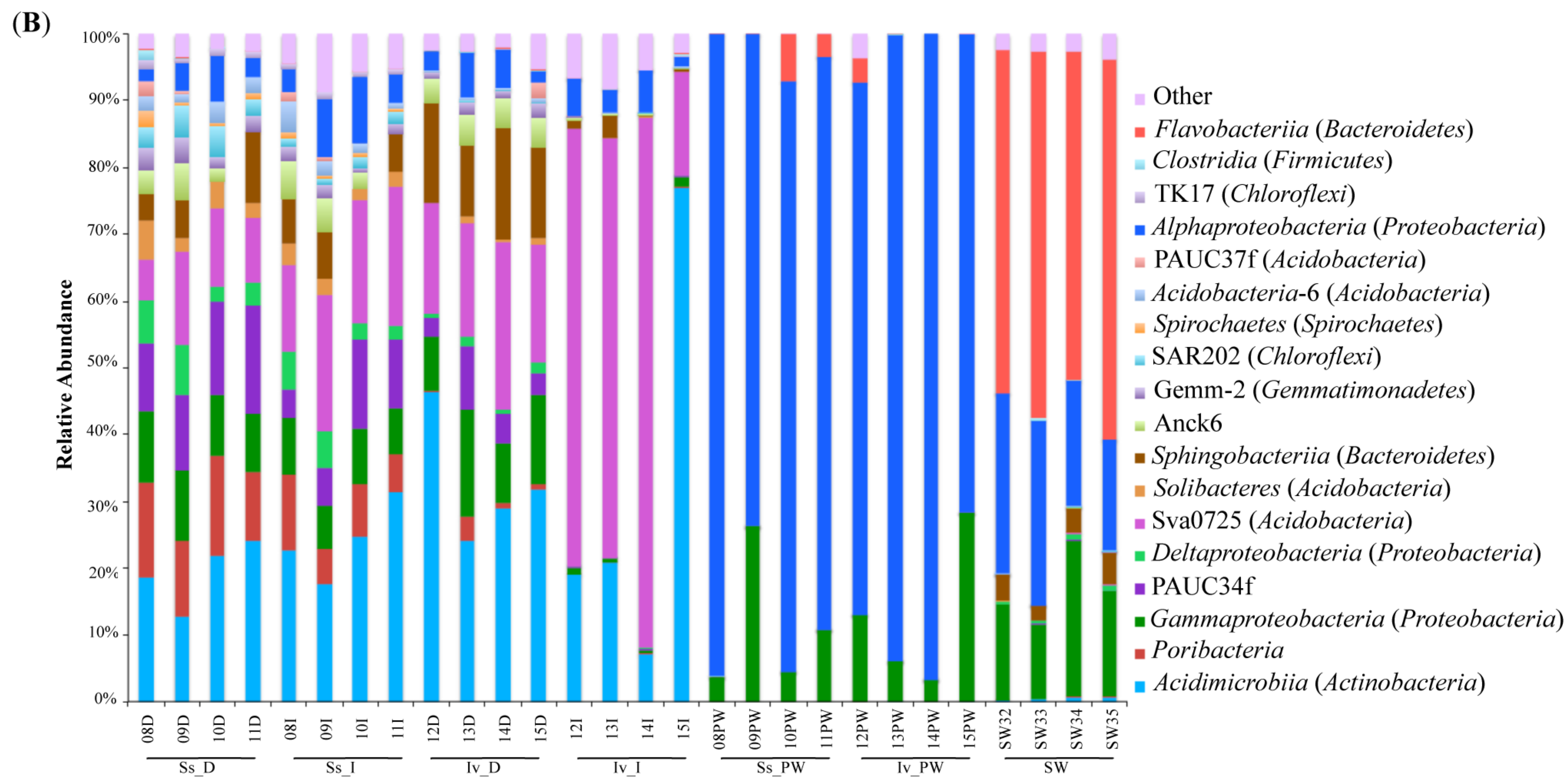

Supplement: Supplementary file 1 [file Presentation_1.ZIP › Supplementary Material/Figure S2.PDF]

(A)

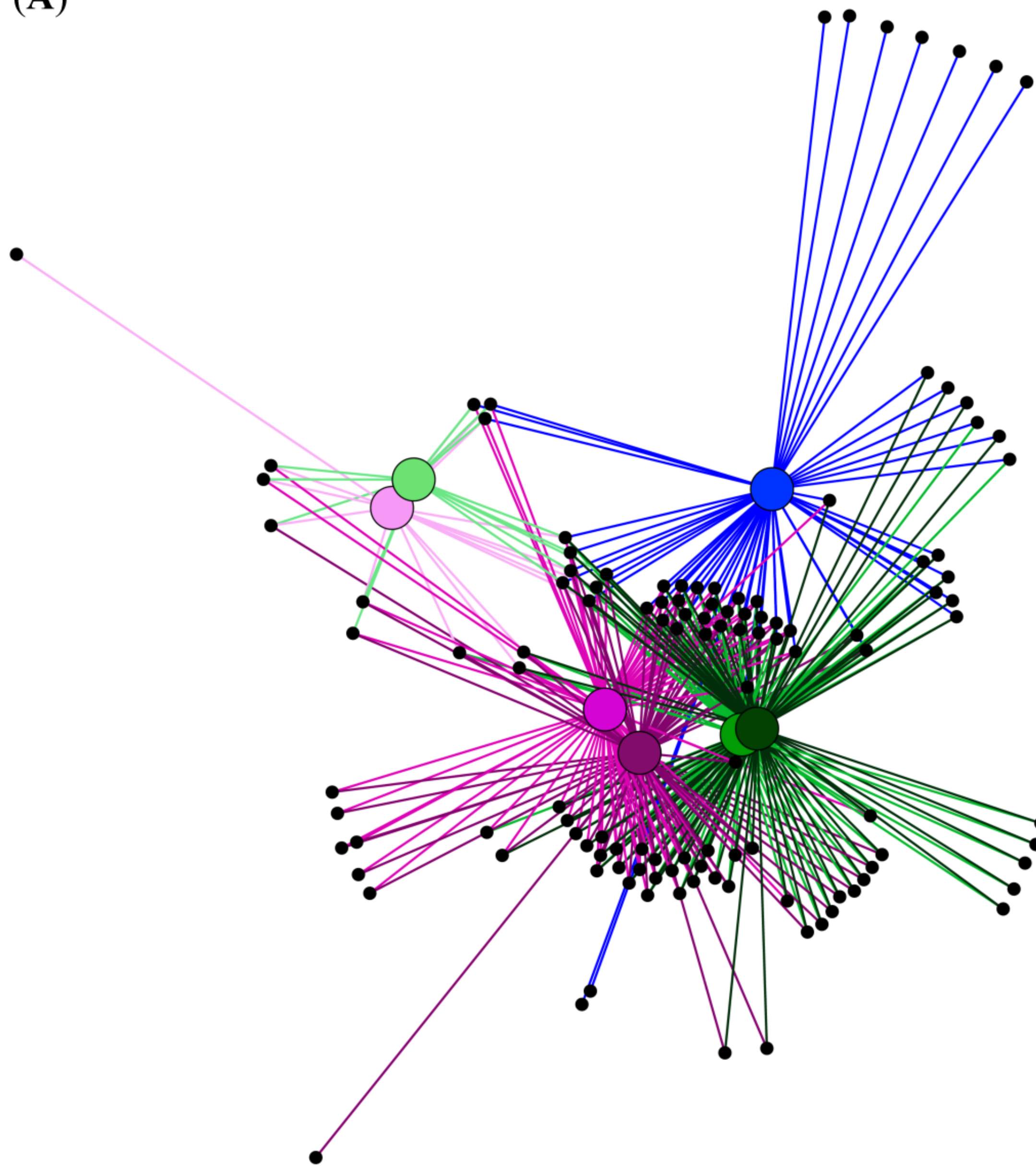

(B)

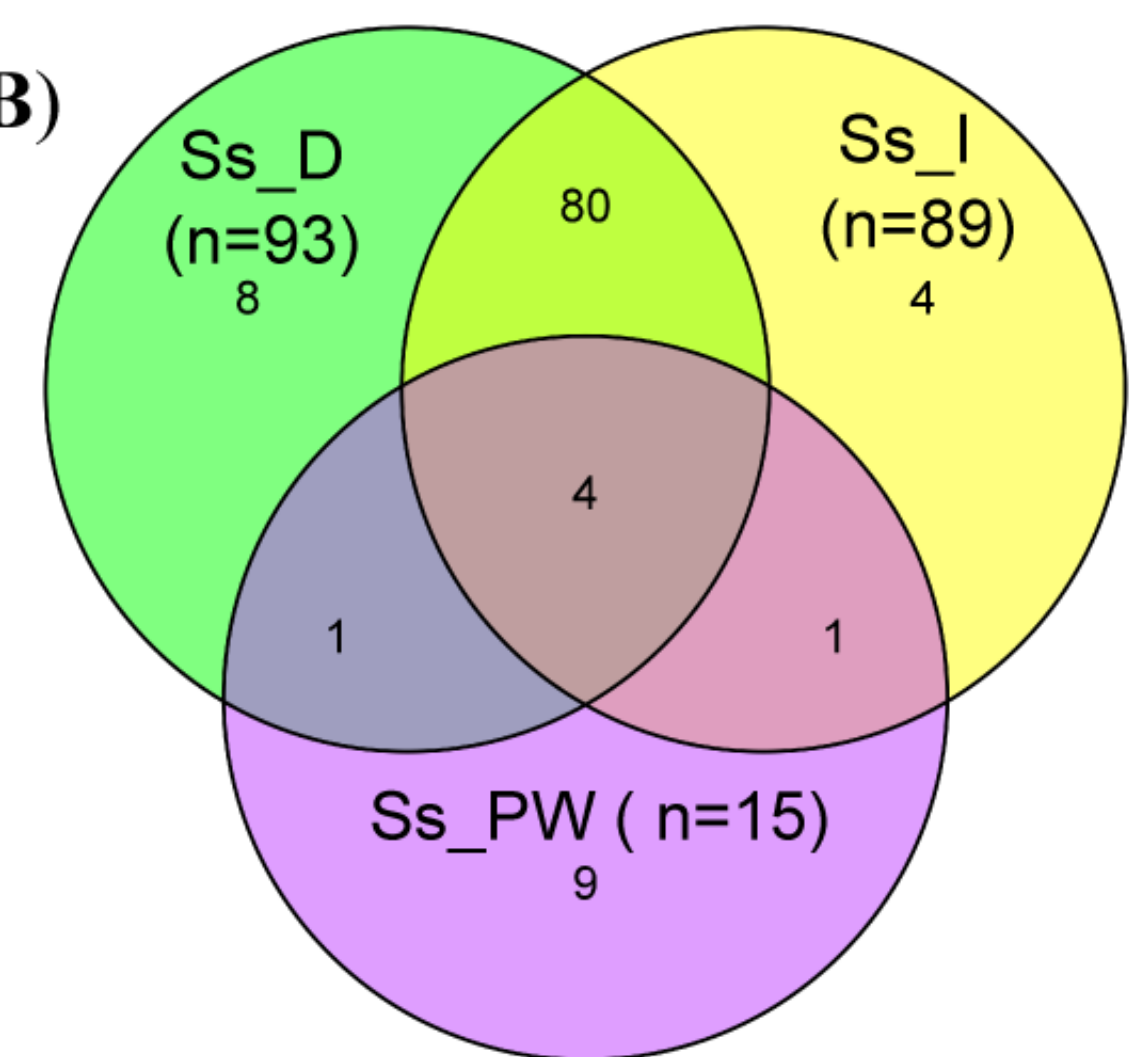

(C)

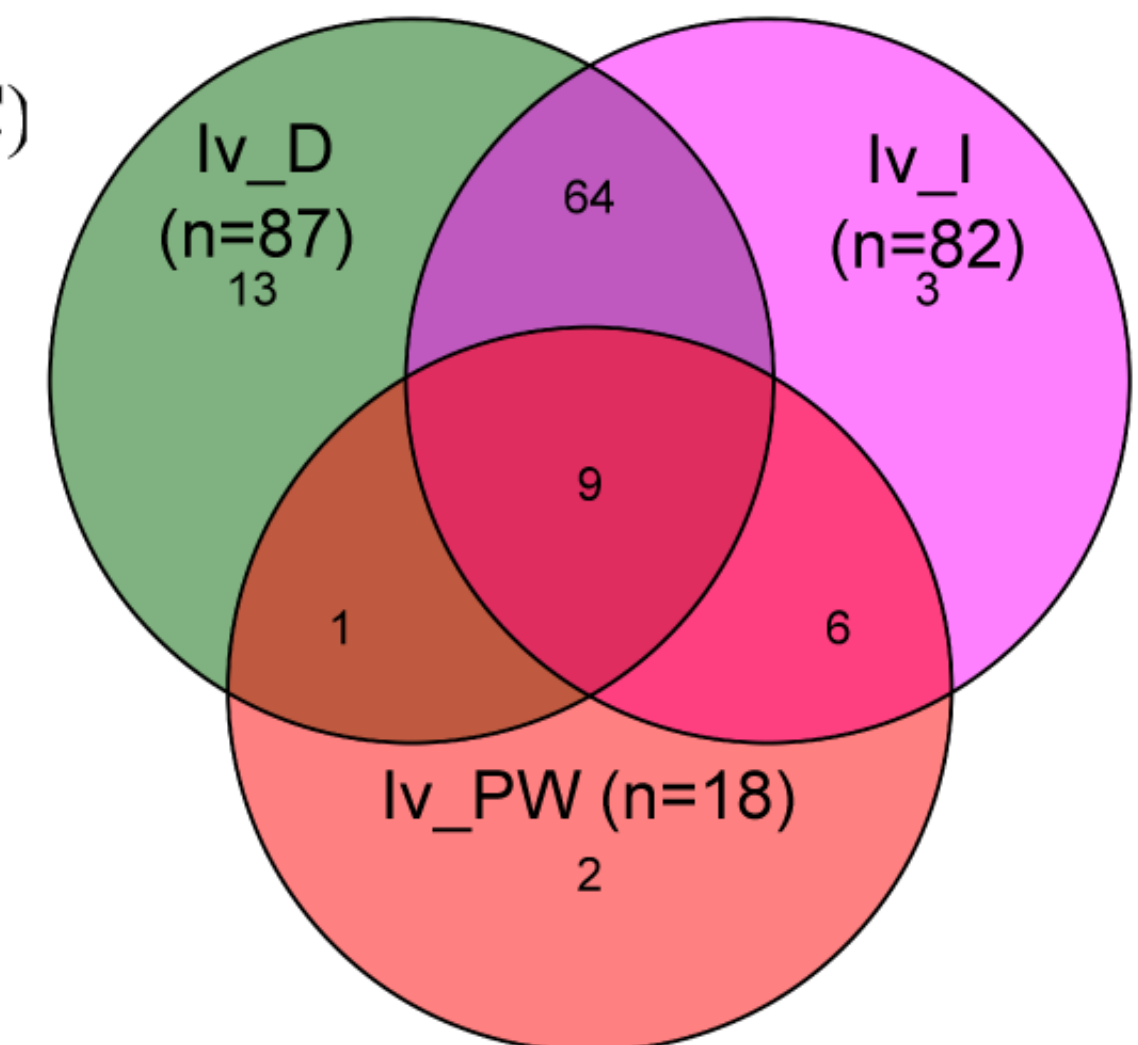

(D)

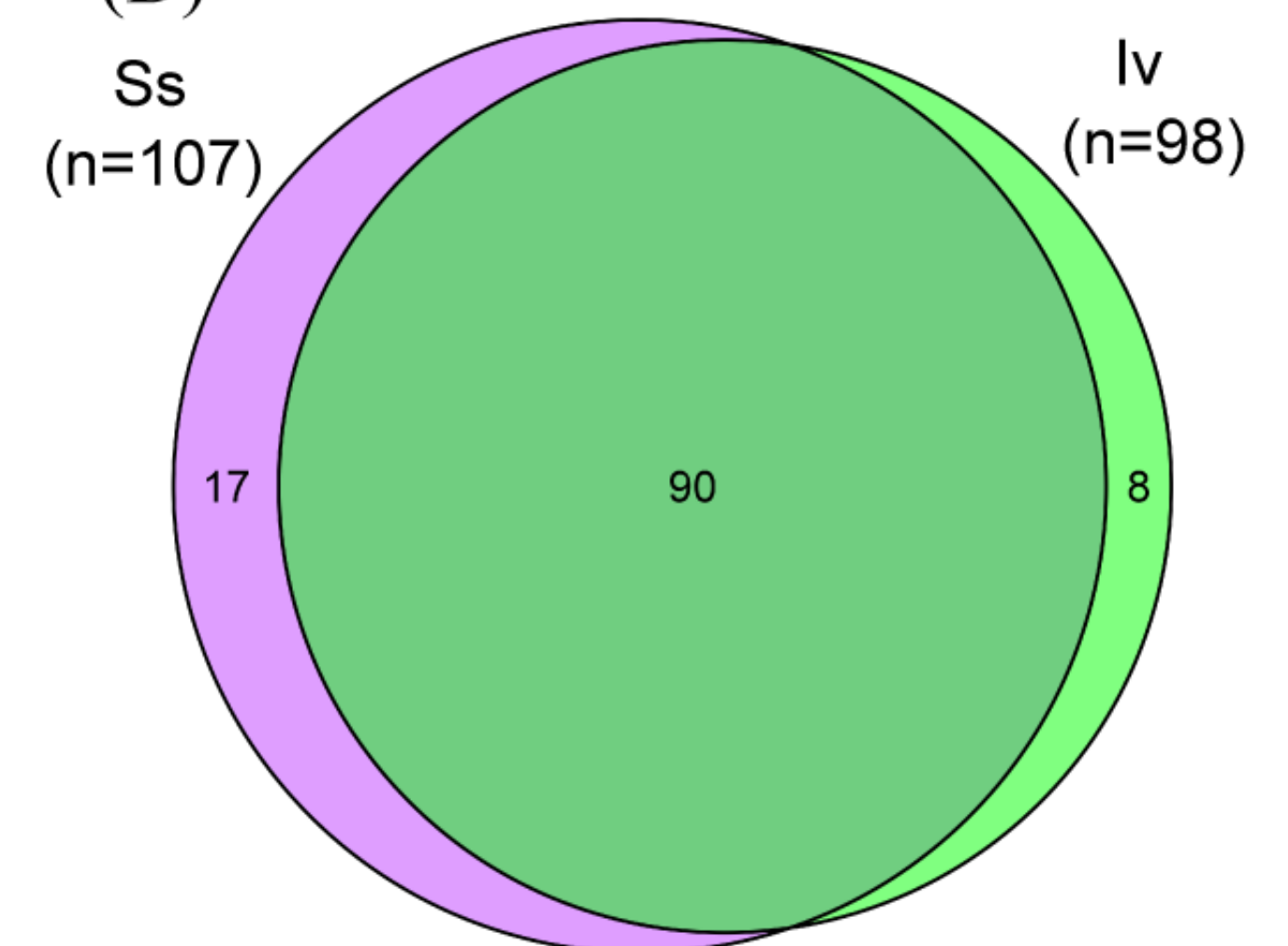

Figure S3

(E)

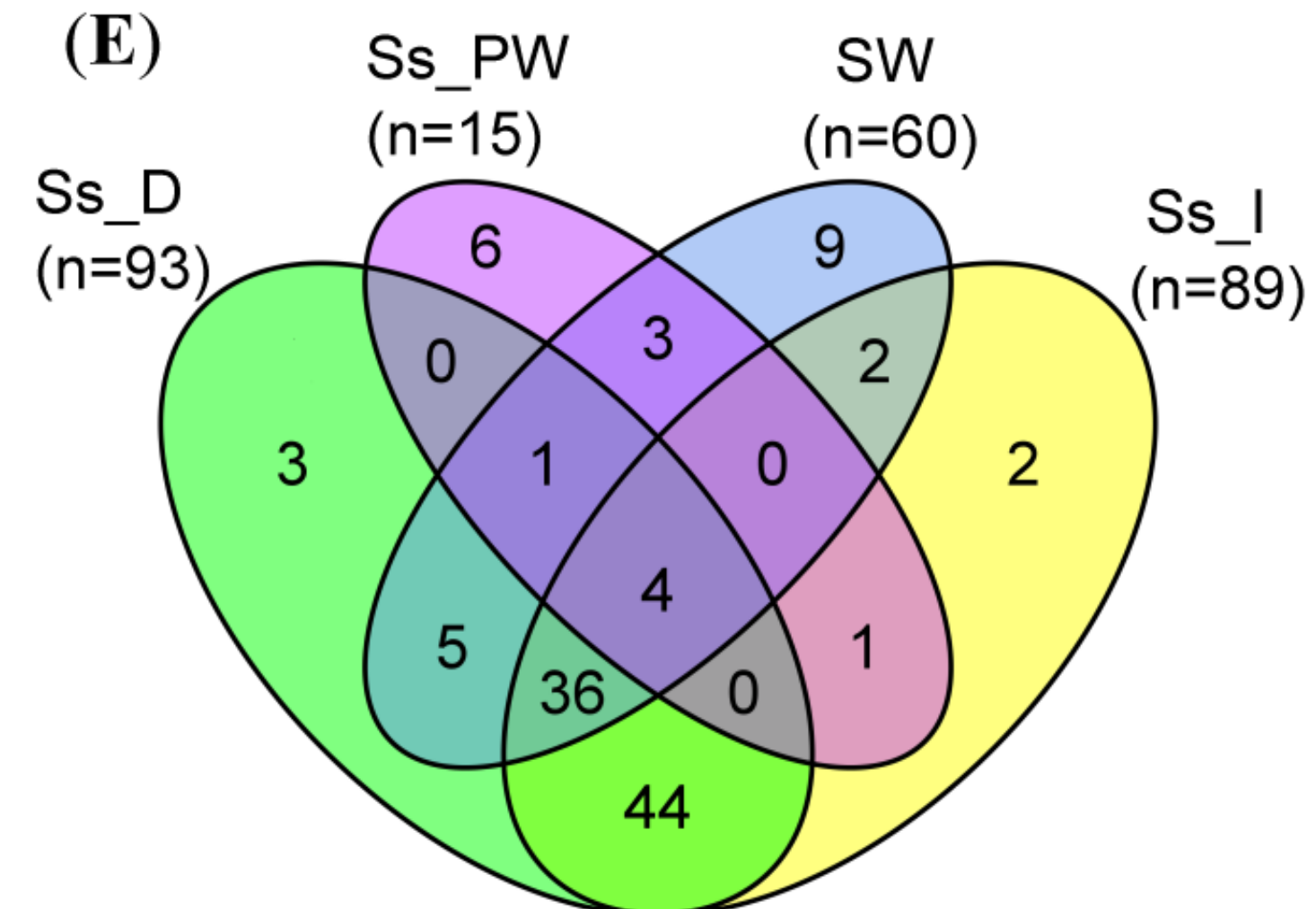

(F)

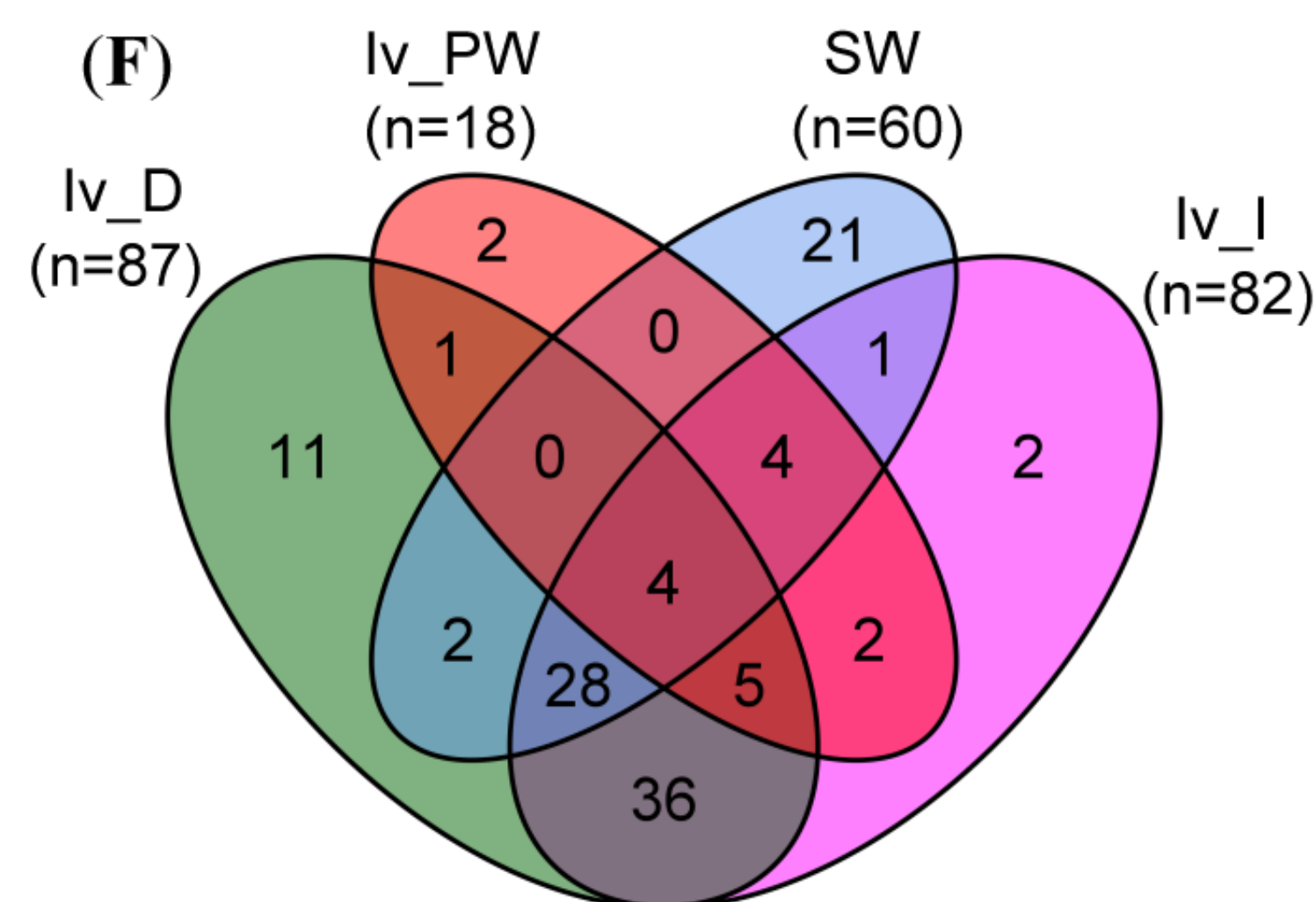

(G)

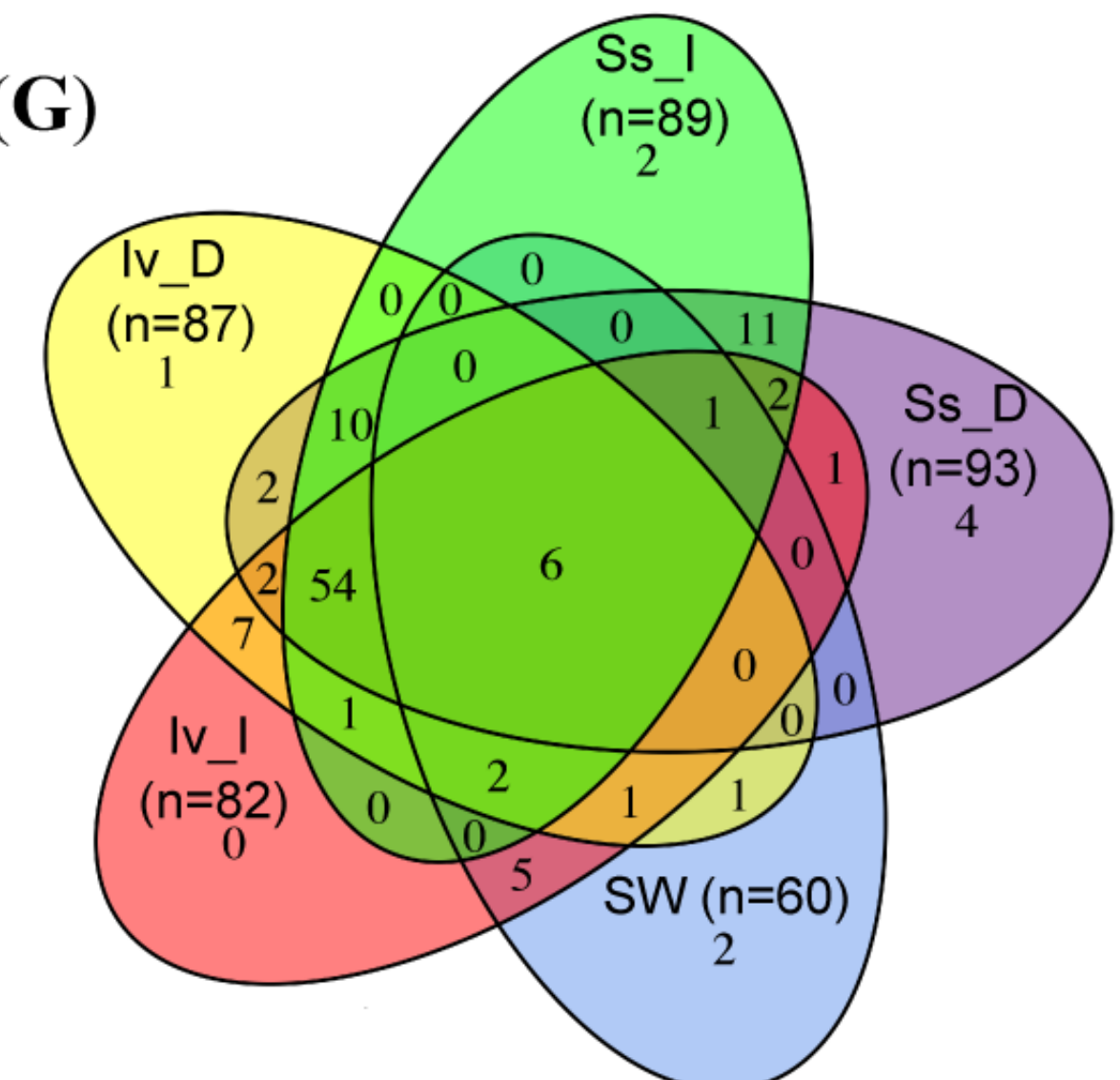

Supplement: Supplementary file 1 [file Presentation_1.ZIP › Supplementary Material/Figure S3.PDF]

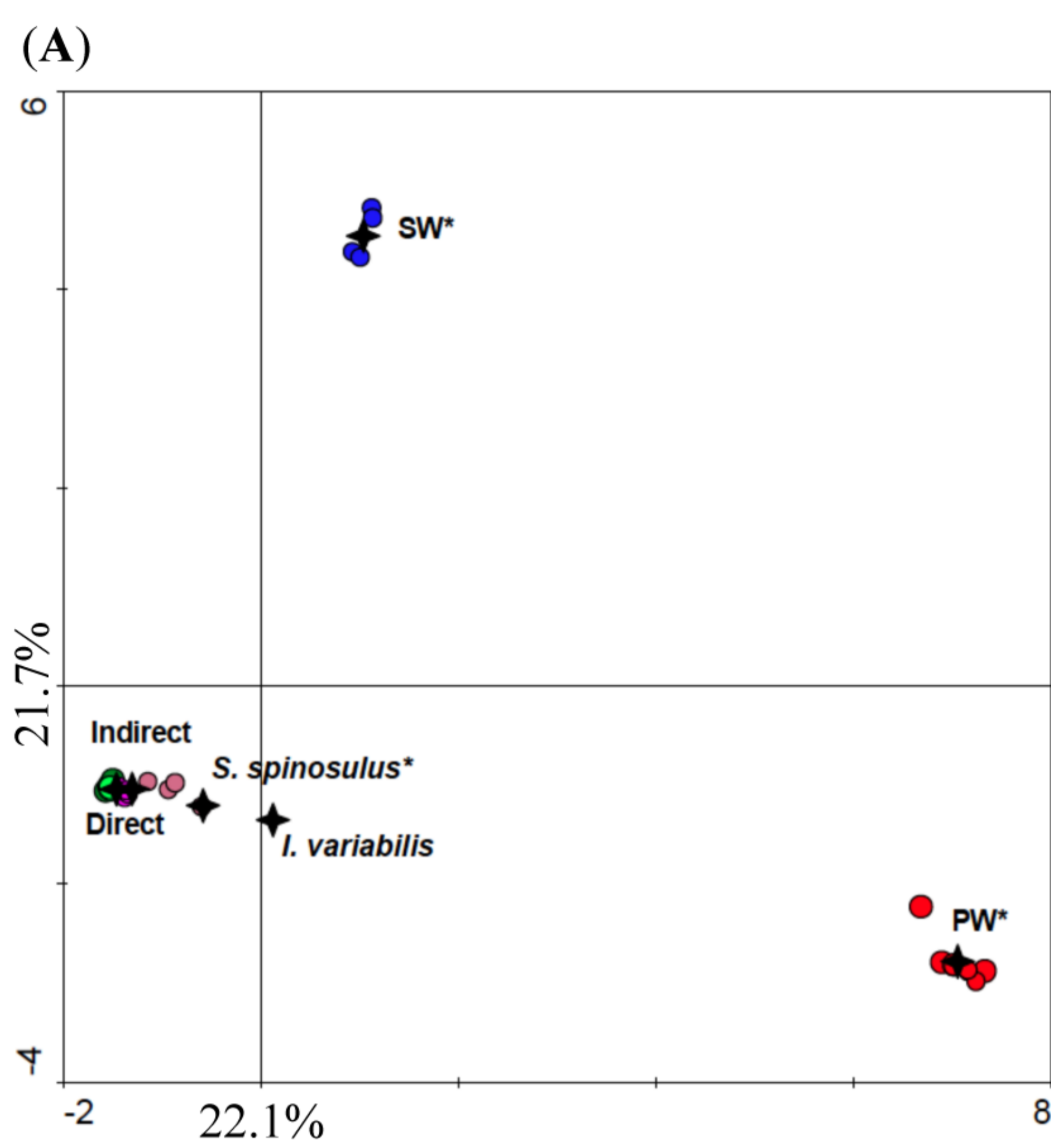

Figure S4

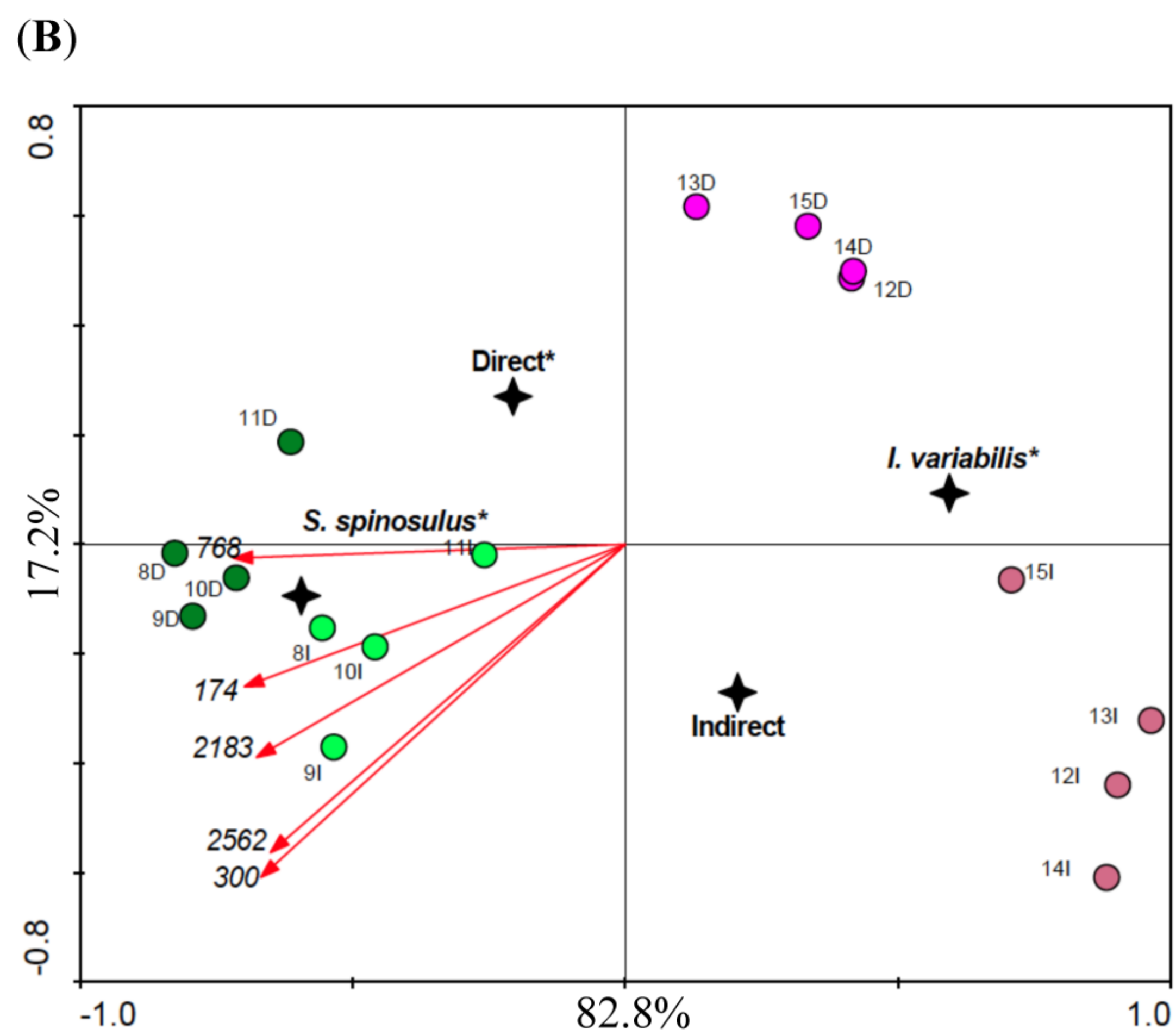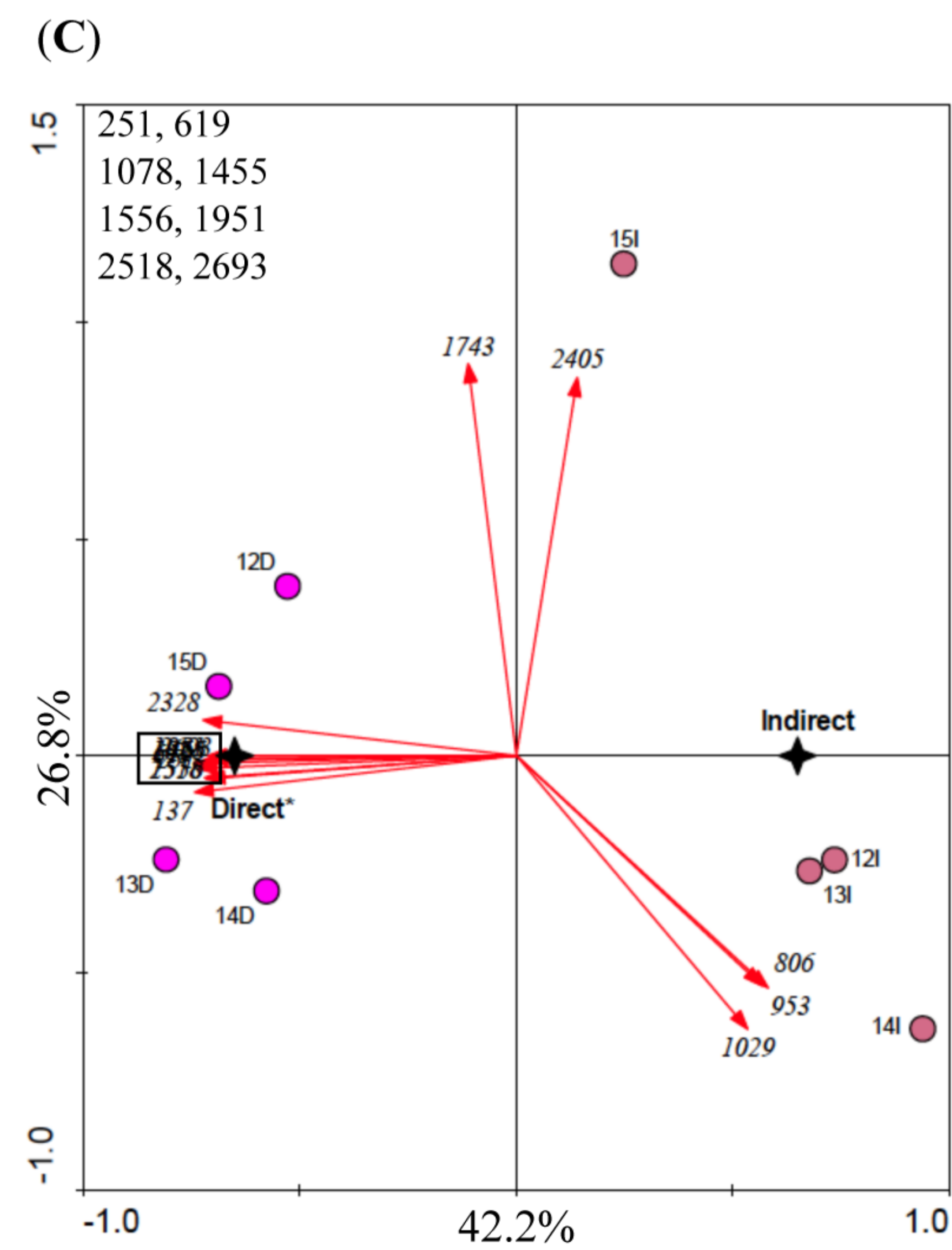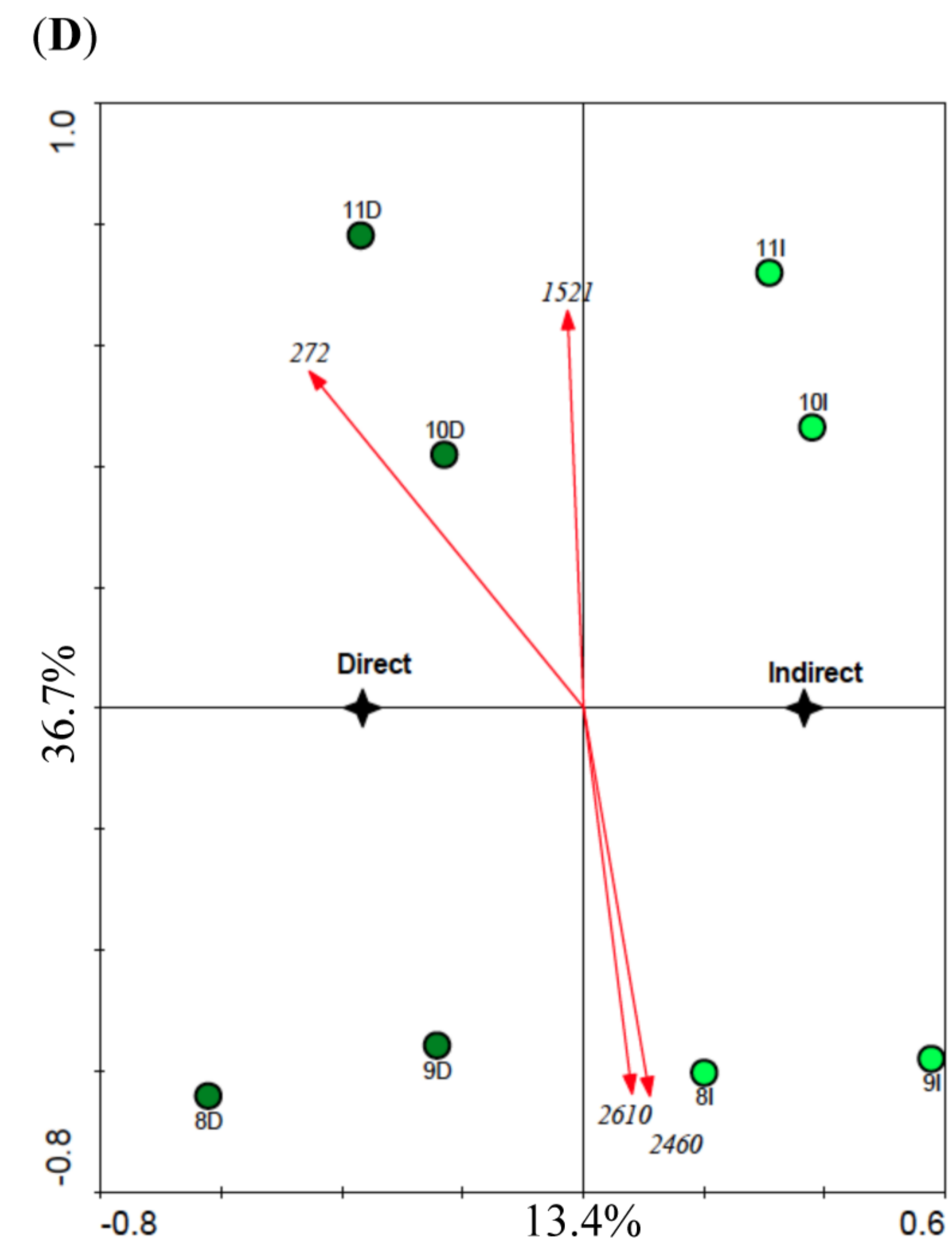

Supplement: Supplementary file 1 [file Presentation_1.ZIP › Supplementary Material/Figure S4.PDF]
